# Use of high-affinity scFv antibody NUsc1 for isolation and sensitive detection of Alzheimer’s-associated amyloid beta oligomers

**DOI:** 10.1016/j.jbc.2026.113499

**Published:** 2026-08-26

**Authors:** Raquel Maria de Campos, Mariane Fávero Carraro, André Luiz Brandão Bitencourt, Joshua J. Jerisha, Navid Jubaer Ayon, Vuslat B. Juska, Nathalia Reges Pinheiro, Elizabeth A. Johnson, Raghad N. Nowar, John P. McGee, Mauro Exequiel Alfaro, Maria Victoria Oberholzer, Vanina Grippo, Erika N. Cline, Kirsten L. Viola, Neil L. Kelleher, Martin Habif, Diana Alicia Jerusalinsky, Gajendra S. Shekhawat, Morgan B. Elmor, James J. Lah, Allan I. Levey, William L. Klein, Adriano Sebollela

**Affiliations:** 1 Department of Neurobiology, Northwestern University, Evanston, Illinois, USA; 2 Department of Biochemistry and Immunology, Ribeirao Preto Medical School, University of São Paulo, São Paulo, Brazil; 3 Departments of Chemistry and Molecular Biosciences, Department of Chemical and Biological Engineering, the Chemistry of Life Processes Institute, The Proteomics Center of Excellence at Northwestern University, Evanston, Illinois, USA; 4 Department of Chemistry, Weinberg College of Arts & Sciences, Northwestern University, Evanston, Illinois, USA; 5 Instituto de Biología Celular y Neurociencia (IBCN), Universidad de Buenos Aires (UBA) and Consejo Nacional de Investigaciones Científicas y Técnicas (CONICET), Ciudad Autónoma de Buenos Aires, Argentina; 6 Instituto de Ciencia y Tecnología “Dr César Milstein” (ICT-Milstein), Consejo Nacional de Investigaciones Científicas y Técnicas (CONICET) – Fundación Pablo Cassará, Ciudad Autónoma de Buenos Aires, Argentina; 7 International Institute for Nanotechnology and NUANCE Center, Department of Materials Science and Engineering, Northwestern University, Evanston, Illinois, USA; 8 Department of Neurology and Goizueta Alzheimer’s Disease Research Center, Emory University, Atlanta, Georgia, USA

**Keywords:** Alzheimer's disease, amyloid β oligomers, conformational antibodies, NUsc1, scFv, ELISA

## Abstract

Alzheimer’s disease (AD) is a progressive neurodegenerative disorder in which soluble amyloid-beta oligomers (AβOs) play a key role in disease onset and progression. This study further explores NUsc1, a single-chain variable fragment (scFv) antibody with high specificity for neurotoxic AβOs over monomers or fibrils, to selectively isolate and assist characterization of AβO species. NUsc1 binds AβOs with a kinetic dissociation constant under 10 nM, comparable to the affinity of Leqembi for protofibrils. NUsc1 inhibits aggregation of AβOs into protofibrillar and fibrillar structures and blocks AβO binding to cultured hippocampal neurons in a dose-dependent manner, with an IC_50_ of ∼100 nM in experiments using 2.25 ng/μl AβOs. Selectivity and high affinity of NUsc1 enabled immunoprecipitation of AβOs under non-denaturing conditions. Atomic force microscopy revealed globular NUsc1–AβO complexes with a z-height of ∼3 to 4 nm, while free NUsc1 measured ∼1.5 nm. Mass spectrometry revealed a heterogeneous population of NUsc1-isolated AβOs, consistent with a stepwise species assembly, while non-denaturing size-exclusion chromatography revealed species of approximately 70 kDa. In ELISA, phage-bound NUsc1 (pbNUsc1) detected ∼12 pM AβOs, a 33-fold increase in detection sensitivity compared to a commercial anti-Aβ antibody. NUsc1 also detected AβOs from 5xFAD mouse brain extracts, cerebrospinal fluid from AD rats, and, notably, from CSF of human donors, demonstrating direct detection of AD-relevant oligomers in a clinically relevant human biofluid. Collectively, these results highlight NUsc1's promise not only as a powerful research tool for structural and mechanistic studies of AβOs but also as a highly sensitive diagnostic tool for clinical use.

Alzheimer’s disease (AD) is a progressive neurodegenerative disorder and the leading cause of dementia worldwide, characterized by gradual memory loss, cognitive decline, and behavioral impairment (1). A central pathological hallmark of AD is the accumulation of amyloid-beta (Aβ) peptides in the brain, which aggregate into various forms comprising oligomers and extracellular amyloid plaques (2). Aβ oligomers (AβOs), soluble aggregates of Aβ, have emerged as the most neurotoxic species, with increasing evidence implicating them in the early disruption of synaptic function, impairment of neuronal signaling, promotion of synapse degeneration, activation of neuroinflammatory pathways, and promotion of neuronal death, events that precede and possibly drive the onset of cognitive symptoms in AD and ultimately the formation of amyloid plaques (3, 4, 5, 6).

Unlike fibrillar deposits, AβOs are metastable and structurally heterogeneous, making them difficult to isolate and characterize (7). Yet, their early appearance and potent toxicity make these assemblies primary targets for both therapeutic intervention and biomarker development (4). The ability to selectively detect, isolate, and study AβOs is critical for understanding their role in AD pathogenesis and for developing strategies to mitigate their effects.

Recent progress in antibody-based approaches has identified NUsc1 as a promising antibody that selectively targets neurotoxic Aβ oligomers. NUsc1 is a genetically engineered antibody fragment, selected from a phage-displayed human library, which is composed of a scaffold formed by the variable regions of heavy and light IgG chains joined by a short Gly-Ser linker. This fragment antibody format is known as a single-chain variable fragment (scFv) (8). NUsc1 binds AβOs with high selectivity, neutralizing their interaction with neuronal membranes and mitigating associated neurotoxicity in both cellular and animal models (9, 10, 11, 12).

In this study, we demonstrate that NUsc1 offers unique advantages as a research tool. It binds AβOs with nanomolar affinity, stabilizes them against further aggregation, and blocks their interaction with neuronal cells in a dose-dependent manner. We further show that NUsc1 enables the isolation of AβOs in their native state, allowing for successful purification and structural characterization of these entities. Finally, we explore the advantages of using NUsc1 in a phage-bound format as detecting antibody in ELISA with enhanced sensitivity, demonstrating its potential to create more efficient assays for detecting neurotoxic oligomeric species in clinical samples. This approach could ultimately enable early diagnostic applications in AD.

Collectively, our findings position NUsc1 as a versatile platform for targeting, studying, and detecting toxic AβOs, facilitating detailed structural and biochemical analyses and offering opportunities for advancing both therapeutic and diagnostic strategies in AD.

## Results

### Binding affinity of NUsc1 toward Aβ oligomers

It was previously shown in animal models that AAV-mediated brain expression of NUsc1 exerts neuroprotective effects and reverse memory deficits in transgenic animals (10), consistent with findings that NUsc1 can selectively bind to synapse-bound Aβ oligomers (AβOs) (9, 12). To further assess the interaction of NUsc1 with AβOs, we have determined its binding affinity using a biolayer interferometry (BLI) assay. In this assay, NUsc1 was immobilized on the BLI probes to establish a baseline. The NUsc1-probes were then immersed in a solution containing varying concentrations of AβOs, and the optical shift of molecular weight on the probe was measured over time. This allowed the association and dissociation of the species to be monitored in real time.

The dissociation constant (Kd) for the interaction between NUsc1 and AβOs, reflecting their binding affinity, was measured at 8.9 nM, which remained consistent across all experimental replicates and across varying concentrations of analyte (Fig. 1 and Table S1). The nanomolar affinity observed between synthetic AβOs and NUsc1 indicates a strong interaction between the antibody fragment and its target. This indicates that only minimal amounts of NUsc1 are required to achieve a strong and specific association with AβOs, supported by a slow dissociation rate that reflects durable target engagement, an essential characteristic of therapeutic antibodies (13).

**Figure 1:**
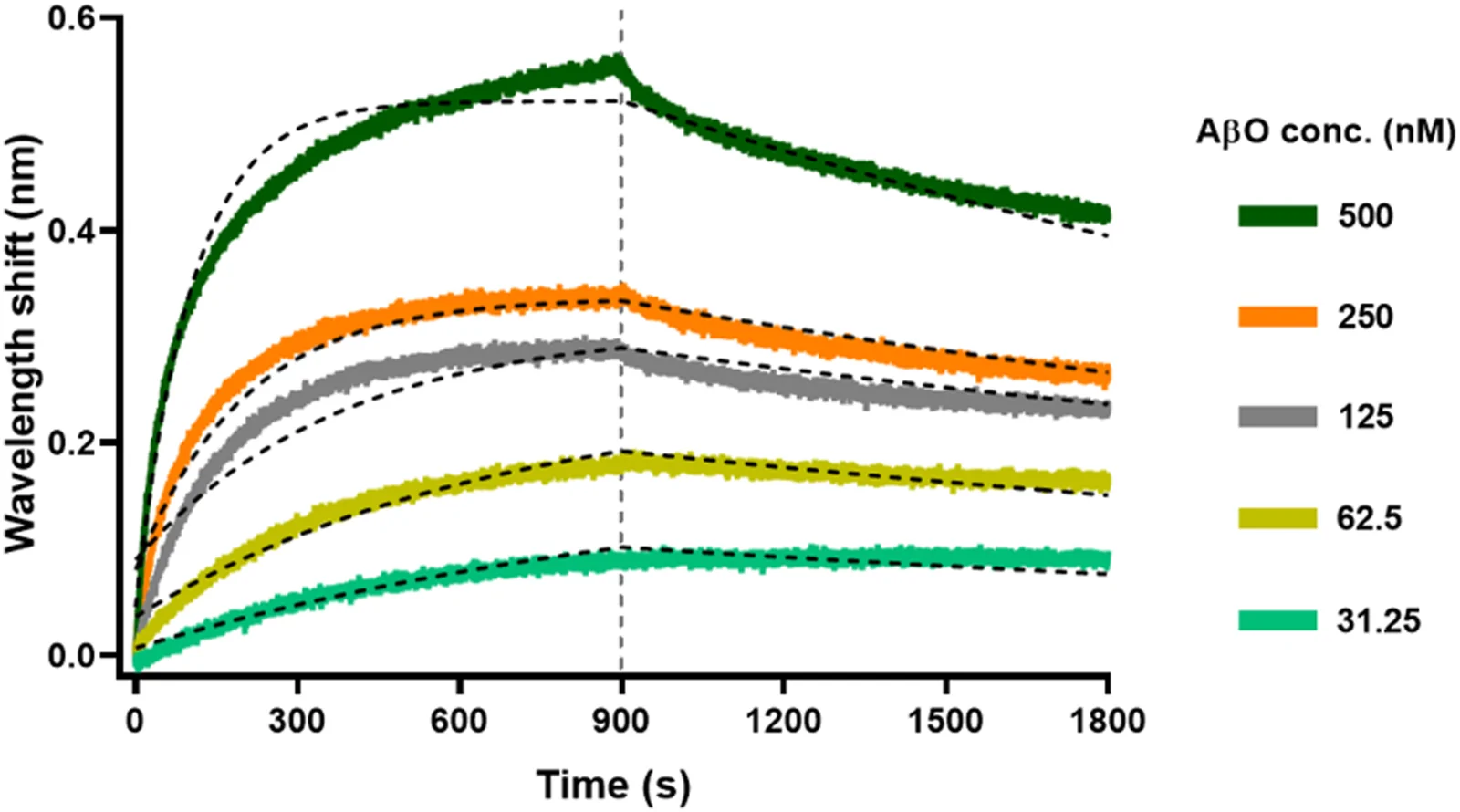
**Bio-layer interferometry analysis of Aβ oligomer binding to NUsc1.** Representative sensorgrams showing binding of AβOs (concentrations ranging from 31.25 to 500 nM) to NUsc1 immobilized on a streptavidin biosensor measured by bio-layer interferometry (BLItz and Octet K2). Association and dissociation phases were monitored for 900 s each. Binding affinity and kinetic parameters were calculated using the Octet BLI Discovery Software. The sensorgram obtained was analyzed using a 1:1 kinetic Langmuir binding model for global data fitting, where Rmax was allowed to vary across analyte concentrations. The changes in optical signal corresponding to molecular binding events were recorded in real-time.

### NUsc1 binding to AβOs blocks continued assembly/conversion to fibrillar species

Next, we examined whether NUsc1 prevents the aggregation of AβOs into larger fibrillar species. This investigation is important for elucidating how NUsc1 may modulate the Aβ aggregation pathway. To assess fibril formation *in vitro*, we first used the thioflavin T (ThT) fluorescence assay, which detects increases in β-sheet content through ThT binding and fluorescence emission at 485 nm, serving as a proxy for the extent of amyloid aggregation (14). In parallel, we used transmission electron microscopy (TEM) to visualize the aggregation endpoints after 20 h.

The ThT fluorescence data demonstrated that NUsc1 completely inhibited the progression of synthetic AβOs into fibrillar forms over a 20-h incubation period (Fig. 2). In contrast, samples lacking NUsc1 exhibited a marked increase in fluorescence signal, indicative of typical Aβ fibril formation (Fig. 2*A*). Transmission electron microscopy (TEM) further substantiated the ThT fluorescence results by visualizing the morphology of Aβ species after 20 h of incubation at 37 °C. In samples lacking NUsc1(Fig. 2*B*), abundant and dense networks of highly branched fibrils were observed, consistent with a mixture of amyloid protofibrils and fibrils (15, 16, 17). These fibrils/protofibrils exhibited diameters of ∼7 to 13 nm, with a mean of 9.6 ± 1.4 nm (n = 24), typical of Aβ fibrillar aggregates (18). In contrast, NUsc1-treated samples displayed a complete absence of fibrillar structures (Fig. 2*C*). Instead, only globular particles were visible, indicating a blockade in fibril formation and the potential neutralization of AβOs species. These morphological differences suggest that NUsc1 inhibits fibril assembly. Together, these findings indicate that NUsc1 effectively impedes the conversion of AβOs into higher-order assemblies, likely by disrupting oligomer–oligomer interactions and interfering with the secondary nucleation-dependent aggregation process (19), which has been determined to play a key role in the mechanism of fibrillogenesis (20).

**Figure 2:**
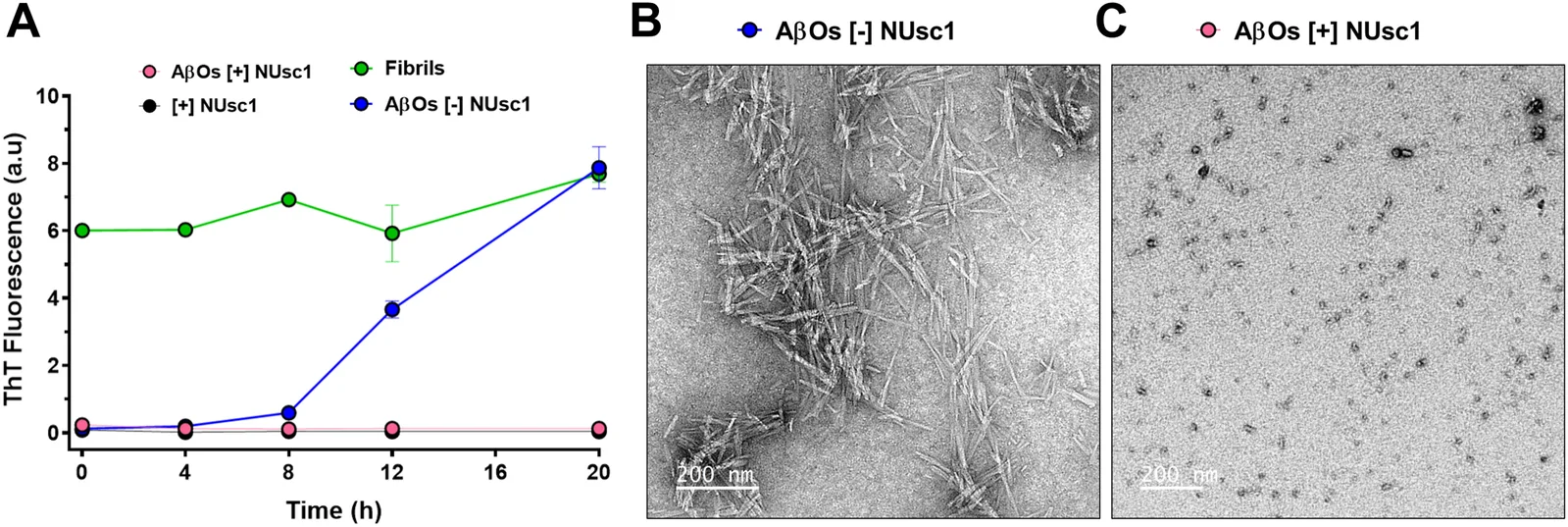
**Inhibition of amyloid protofibril/fibril formation by scFv NUsc1.***A*, AβO solutions (10 μM) were incubated at 37 °C for 20 h in the presence or absence of scFv NUsc1 (5 μM). Amyloid fibril formation was monitored by Thioflavin T (ThT) fluorescence, with preformed Aβ fibrils used as a positive control. Transmission electron microscopy (TEM) images of endpoint samples (20 h) reveal morphological differences in AβO aggregates in (*B*) the absence and (*C*) the presence of NUsc1 treatment. Fibril thickness was measured in ImageJ using the set scale method. Six individual fibrils or protofibrils were selected, and four perpendicular width measurements were taken along each, then averaged to determine the mean diameter.

### Dose-dependent inhibition of neuronal AβO binding by NUsc1

To investigate the dose-dependent inhibitory capacity of NUsc1 on synthetic AβO binding to neurons, we assessed its ability to neutralize AβO interactions with primary neuronal cells across a range of concentrations. Mature rat hippocampal neuronal cultures (DIV24) were treated with vehicle control, cross-linked AβOs alone, or pre-incubated mixtures of AβOs and NUsc1 at varying molar ratios. Cross-linked AβOs were used based on established protocols (21), as cross-linking stabilizes oligomers in a soluble, neurotoxic conformation. This is particularly useful for live-cell assays at 37 °C, where membrane interactions can promote fibril formation (22).

The results show that NUsc1 effectively prevents AβO binding (Fig. 3), with efficacy increasing significantly at ratios of 3:1 (AβOs:NUsc1) and greater, as demonstrated in Figure 3, *C*–*F*. In contrast, AβOs alone produced a strong membrane-associated signal, while vehicle controls showed negligible signal (Fig. 3, *A* and *B*). Quantification revealed an exponential decay in AβO binding intensity (Fig. 3*G*), with NUsc1 achieving ∼85% inhibition at a 1:1 ratio and almost 100% inhibition at 1:2, indicating strong dose-dependent neutralizing activity. However, effectiveness was markedly reduced at a 10:1 ratio, emphasizing that precise AβOs:NUsc1 stoichiometry is critical for selective binding and optimal inhibitory function. Results were consistent across two independent biological replicates, with five neuronal cells analyzed per coverslip in each treatment condition (n = 5 per condition). The calculated IC_50_ of 0.1 μM demonstrates NUsc1’s high potency at submicromolar concentrations, supporting its suitability for *in vivo* applications (10, 23, 24). Interestingly, the IC_50_ is higher than the Kd, as expected for monovalent antibodies, likely reflecting the lack of avidity and the need for multiple NUsc1 molecules to block each AβO molecule effectively (25) and, in part, the dependence of IC_50_ on ligand concentration under the assay conditions. These data suggest that NUsc1 likely interferes with oligomer conformation or receptor engagement domains, reinforcing its promise as a therapeutic candidate to block early synaptic toxicity in Alzheimer’s disease.

**Figure 3:**
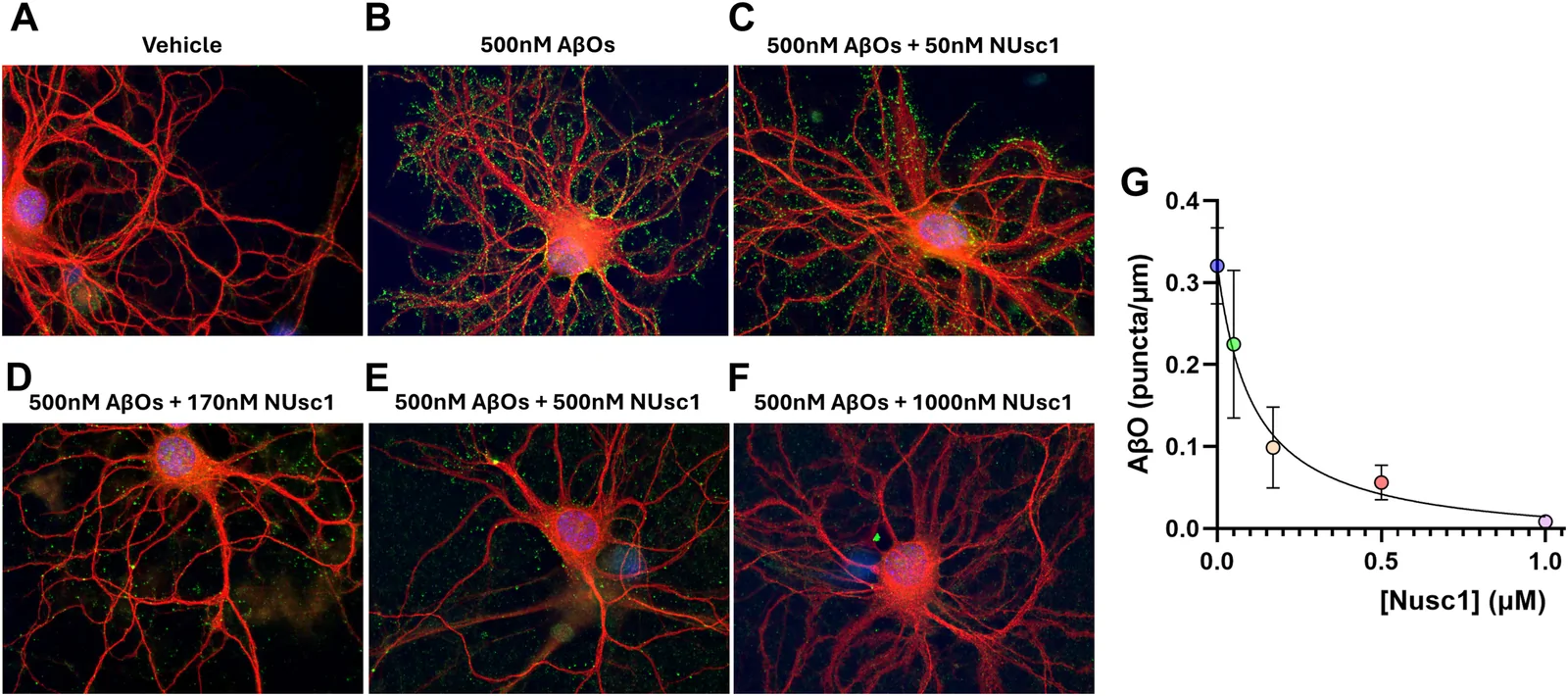
**Neutralization of AβOs by NUsc1 in primary cultured hippocampal neurons.** The cells were treated for 1 h at 37 °C, followed by washing and fixation with formaldehyde at concentrations ranging from 1.4% to 3.7% for a total duration of 20 min. After fixation, coverslips containing the cells were briefly washed with 1X PBS and blocked with 10% normal goat serum (NGS) for 1 h at room temperature on an orbital shaker. Subsequently, the coverslips were incubated overnight at 4 °C in an orbital shaker with primary antibodies: NU2 (1:2000 dilution) and MAP2 (1:10,000 dilution), both diluted in blocking buffer. Following the incubation, the coverslips were washed three times for 10 min each with 1X PBS. They were then incubated with secondary antibodies: anti-mouse-488 conjugate and anti-chicken-568 conjugate, both at a 1:2000 dilution in a 1% NGS solution, for 3 h at room temperature in the dark on an orbital shaker. After a final round of washes, coverslips were mounted on glass slides using a drop of DAPI solution and left to dry overnight at room temperature in the dark. The mounted samples were then imaged using a DM6B microscope with a 63x objective lens. *A–F*, representative single-cell images showing a dose-dependent reduction in AβO binding with increasing concentrations of NUsc1 compared to vehicle-treated controls. *G*, quantification of AβO puncta per micron of dendritic length was performed using ImageJ from n = 10 images per condition. A dose–response curve was generated using nonlinear regression to a sigmoidal model with a variable Hill slope in GraphPad Prism.

### Biochemical isolation of the NUsc1/AβO complex

To further investigate the interaction between NUsc1 and AβOs, we evaluated whether NUsc1 could immunoprecipitate AβO species from solution under non-denaturing conditions using His-tag magnetic beads. Building on previous evidence that NUsc1 selectively targets neurotoxic AβOs, preferentially assemblies larger than 50 kDa (9, 10, 12), we aimed to determine whether NUsc1 forms a stable complex with AβOs that could be isolated and further analyzed.

In-bead ELISA and Western blot analyses demonstrated that NUsc1 effectively immunoprecipitates AβO species from synthetic preparations (Fig. 4). In ELISA, NUsc1-bound samples exhibited a 14-fold increase in AβO signal relative to that obtained using a control scFv targeting an unrelated epitope (*p* = 0.0404, unpaired two-tailed Student’s *t* test), indicating selective binding (Fig. 4*B*). Western blotting of eluate fractions obtained under native conditions (imidazole-based elution) revealed co-detection of AβOs (probed with NU2) and NUsc1 (detected using anti-Myc antibody 9E10), confirming their association within the same molecular complex (Fig. 4, *C* and *D*). No AβO signal was observed in control conditions, excluding nonspecific binding to the beads. Western blot analysis also reveals a variety of AβO species isolated by NUsc1, appearing as a smear reaching up to ∼130 kDa (Fig. 4*D*). Furthermore, AβOs remained bound following multiple wash steps, indicating that the NUsc1–AβO interaction is stable under physiological buffer conditions. Together, these findings demonstrate that NUsc1 forms a stable and isolatable complex with synthetic AβOs, supporting its application in the selective enrichment and structural interrogation of toxic AβO species under native-like conditions.

**Figure 4:**
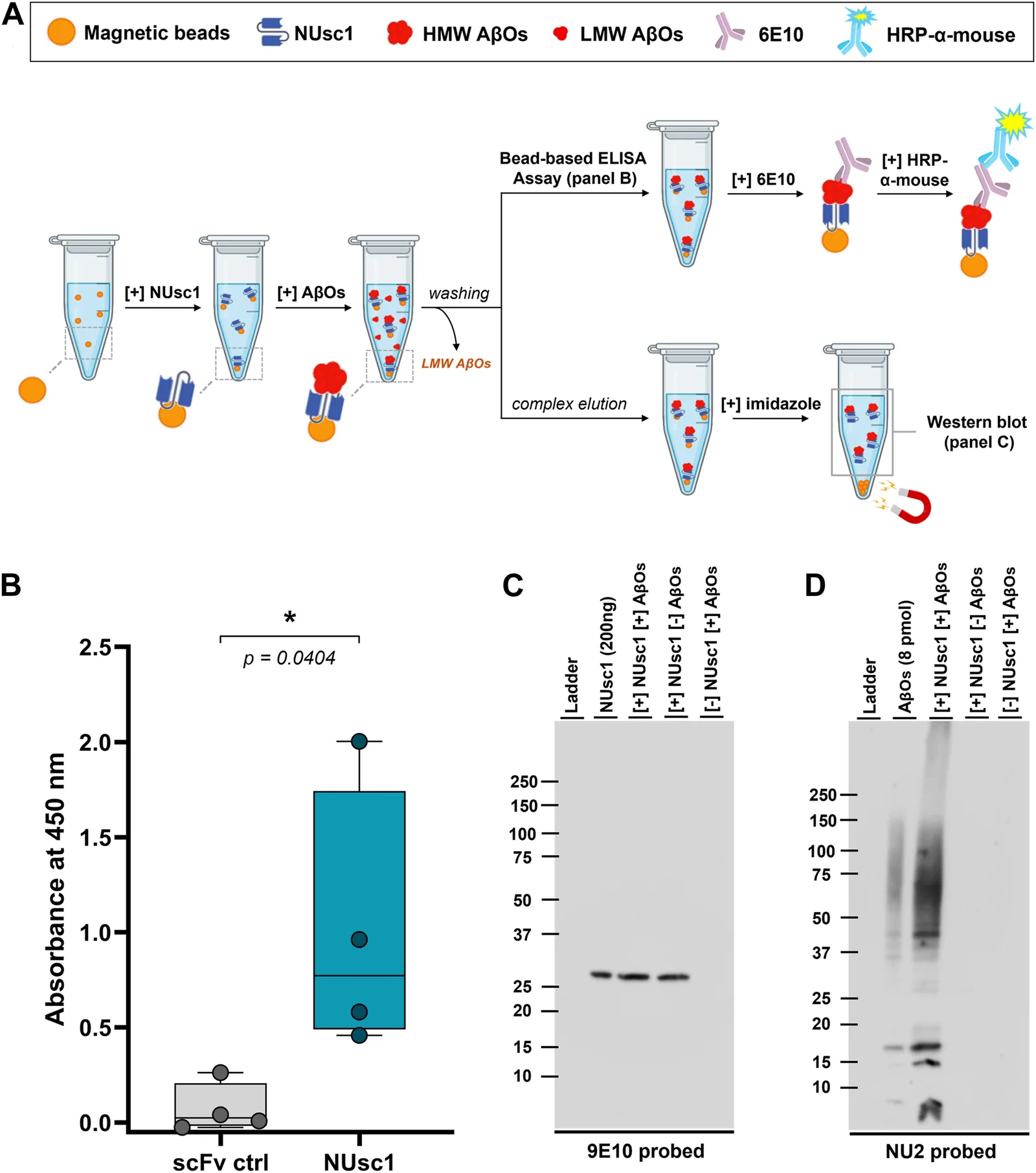
**Isolation of the NUsc1-AβO complex.***A*, schematic representation of the pull-down strategy. Cobalt-coated magnetic beads were incubated with a NUsc1 solution and blocked with a 0.75% BSA solution. AβOs were incubated with magnetic beads functionalized with either NUsc1 or a control scFv. *B*, unbound AβOs were washed away, and an in-bead ELISA was performed by probing the complex with anti-Aβ antibody 6E10, followed by an anti-mouse antibody conjugated with HRP. Approximately 20% of the beads were analyzed by bead-based ELISA using anti-Aβ antibody (6E10) for detection. The data shown corresponds to four independent replicates. ∗ Unpaired *t* test, *p* = 0.0404. *C-D* Alternatively, proteins bound to the remaining beads were eluted with imidazole and analyzed by Western blot using antibodies against c-myc tag (9E10) and NU2 (anti-AβOs). Stock solutions of AβOs and NUsc1 were included as controls.

### Atomic force microscopy and top-down mass spectrometry of the NUsc1–AβO complex

To assess the size and aggregation state of NUsc1-isolated AβO species, we employed atomic force microscopy (AFM) and native top-down mass spectrometry (nTDMS), both of which have proven effective in studying aggregates associated with other neurodegenerative diseases (26, 27, 28).To enable structural analysis, imidazole eluates containing NUsc1 previously incubated with either vehicle or AβOs were deposited onto freshly cleaved mica and prepared for imaging by tapping mode atomic force microscopy (AFM). In parallel, NUsc1-functionalized M-270 (epoxy) beads—more amenable to downstream mass spectrometry—were used to isolate synthetic AβOs (Fig. S3), which were subsequently analyzed by Individual Ion Mass Spectrometry (I^2^MS).

AFM imaging of the NUsc1–AβO complex revealed a heterogeneous population of aggregates, predominately round-shaped with individual species displaying z-heights in the range of ∼3 to 4 nm (Fig. 5*B*). In contrast, free NUsc1 appeared as uniform, round particles with an average z-height of ∼1.5 nm, consistent with its monomeric form (Fig. 5*A*). These topographical features suggest that the AFM-detected species likely correspond to AβO species in the low-nanometer size range, compatible with small, globular oligomers. Complementary analysis using individual ion mass spectrometry (I^2^MS) revealed a multispecies distribution, with synthetic AβO species ranging from ∼15 to 45 kDa and regularly spaced peaks differing by approximately 4.5 kDa, consistent with the theoretical mass of a single Aβ_42_ monomer (4514 Da), an indicative of stepwise oligomerization (Fig. 5*E*). The observed pattern suggests that higher-order oligomers may partially disassemble or fragment, likely during alkaline elution or electrospray ionization, the latter of which has been reported in previous studies (29). These high-resolution observations prompted further investigation using complementary non-denaturing techniques to better estimate the apparent molecular size and assess the oligomerization state of Aβ under native conditions.

**Figure 5:**
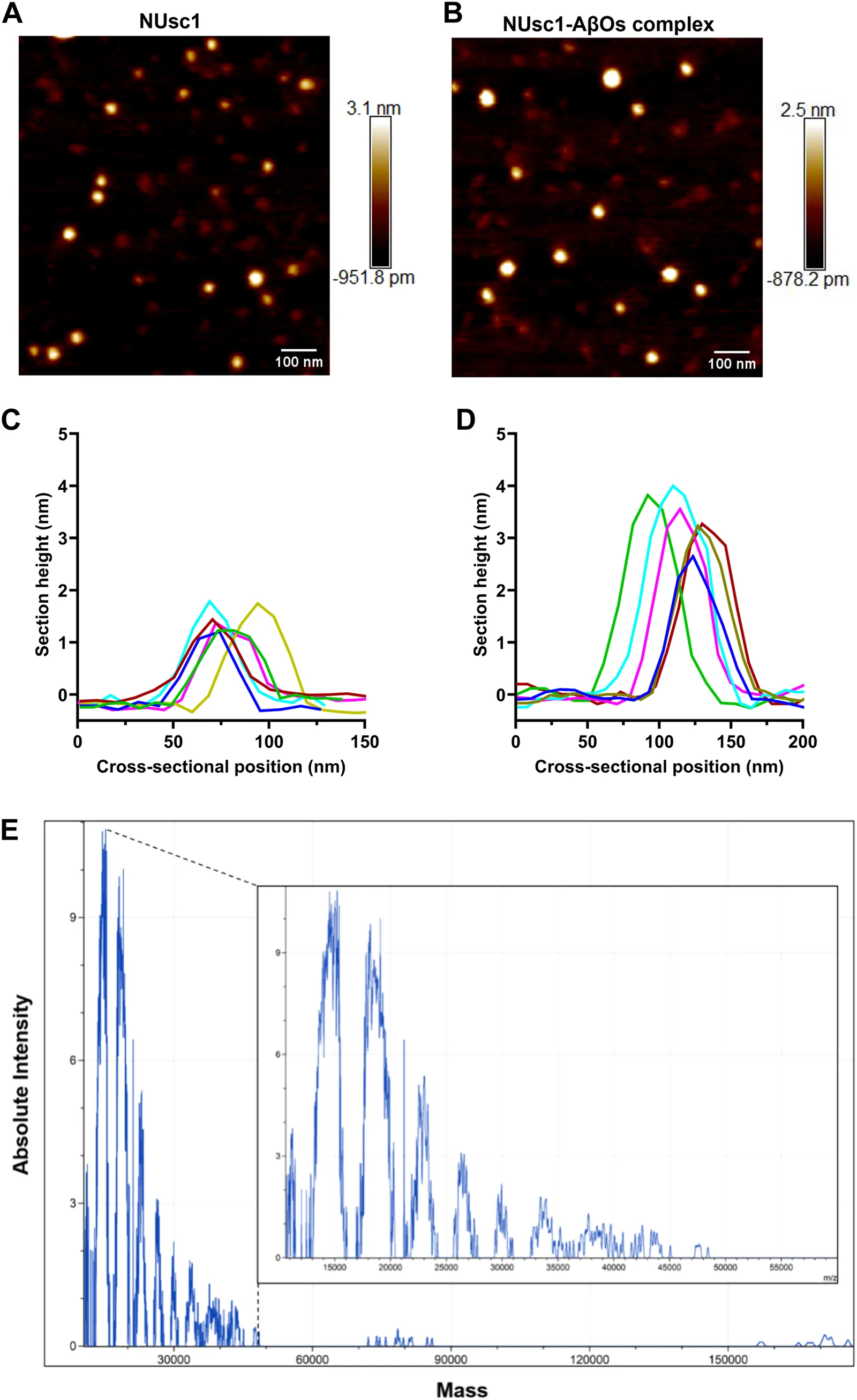
**Size of NUsc1-Bound AβO Species determined by AFM and I^2^MS.** Atomic force microscopy of the (*A*) free NUsc1 reveals predominantly globular structures with relatively uniform size, in contrast to (*B*) NUsc1–AβO complexes. Color scales represent relative z-height in nanometers and are shown independently for each image. *C* and *D*, representative cross-sectional height profiles extracted from AFM images of (*C*) free NUsc1 and (*D*) NUsc1–AβO complexes. Cross-sections of six individual molecules per condition were selected, with lengths (150–200 nm) chosen to capture the full local environment surrounding each molecule. The vertical (height) scale was kept consistent across all profiles to enable direct comparison of molecular heights, whereas lateral dimensions were not directly compared due to varying cross-section lengths (Fig. S4). *E*, individual ion mass spectrometry (I^2^MS) spectra in the mass domain display native Aβ oligomers immunopurified using the NUsc1 antibody. The y-axis shows absolute intensity, directly reflecting oligomer abundance. High-resolution insets provide detailed views of selected oligomeric species.

### Size and oligomerization state of AβO species isolated by NUsc1

Next, we assessed the apparent molecular mass and oligomerization state of the oligomer-antibody complexes using non-denaturing techniques. Eluates obtained from the His-tag-based NUsc1-functionalized beads immunoprecipitation were subjected to size filtration using 100 kDa molecular weight cut-off filters, followed by transfer to nitrocellulose membranes for immunoblotting. The abundance of NUsc1 and AβOs was assessed in the input, retentate and filtrate fractions. In a complementary approach, retentates were analyzed by size-exclusion chromatography (SEC), following the fluorescence emission of Tryptophan residues of NUsc1. This comparison enabled estimation of the apparent size of the AβO species targeted by NUsc1.

Immuno-dot blot analysis demonstrated that the NUsc1/AβO complex is retained by a 100 kDa molecular weight cut-off filter, indicating a complex size exceeding 100 kDa, as both NUsc1 and AβOs were mainly detected in the retentate (Fig. 6, *A* and *B*). The attenuated NUsc1 signal detected in the filtrate (Fig. 6*B*) indicates a smaller fraction of immobilized NUsc1 eluted as free antibody during immunoprecipitation. Size-exclusion chromatography (SEC) of the NUsc1/AβO complex revealed two major peaks corresponding to apparent molecular weights of approximately 86 kDa and 112 kDa, whereas NUsc1 alone eluted at an apparent size of 24 kDa (Fig. 6*C*). The difference in size between the complex and free NUsc1 shows that the isolated Aβ-oligomers range from approximately 62 to 88 kDa. These data indicate that NUsc1 selectively captures AβO species larger than 60 kDa. Based on the estimated molecular weights, the predominant AβO species likely correspond to mid-to high-order oligomers. It is important to note that NUsc1 presented an apparent molecular weight lower than its theoretical mass (29.5 kDa), which may influence the apparent size estimation of the NUsc1/AβO complexes. Additionally, MWCO cutoffs are not absolute, and a fraction of molecules can be retained even if they fall outside the nominal size range, depending on their shape and behavior in solution, which may contribute to the retention of the ∼86 kDa species. Due to the limited sensitivity of the technique, the SEC chromatogram likely reflects only the most abundant AβO species, potentially omitting less abundant oligomeric forms. The current results align with earlier observations reported by Sebollela *et al.* (2017), further supporting their relevance.

**Figure 6:**
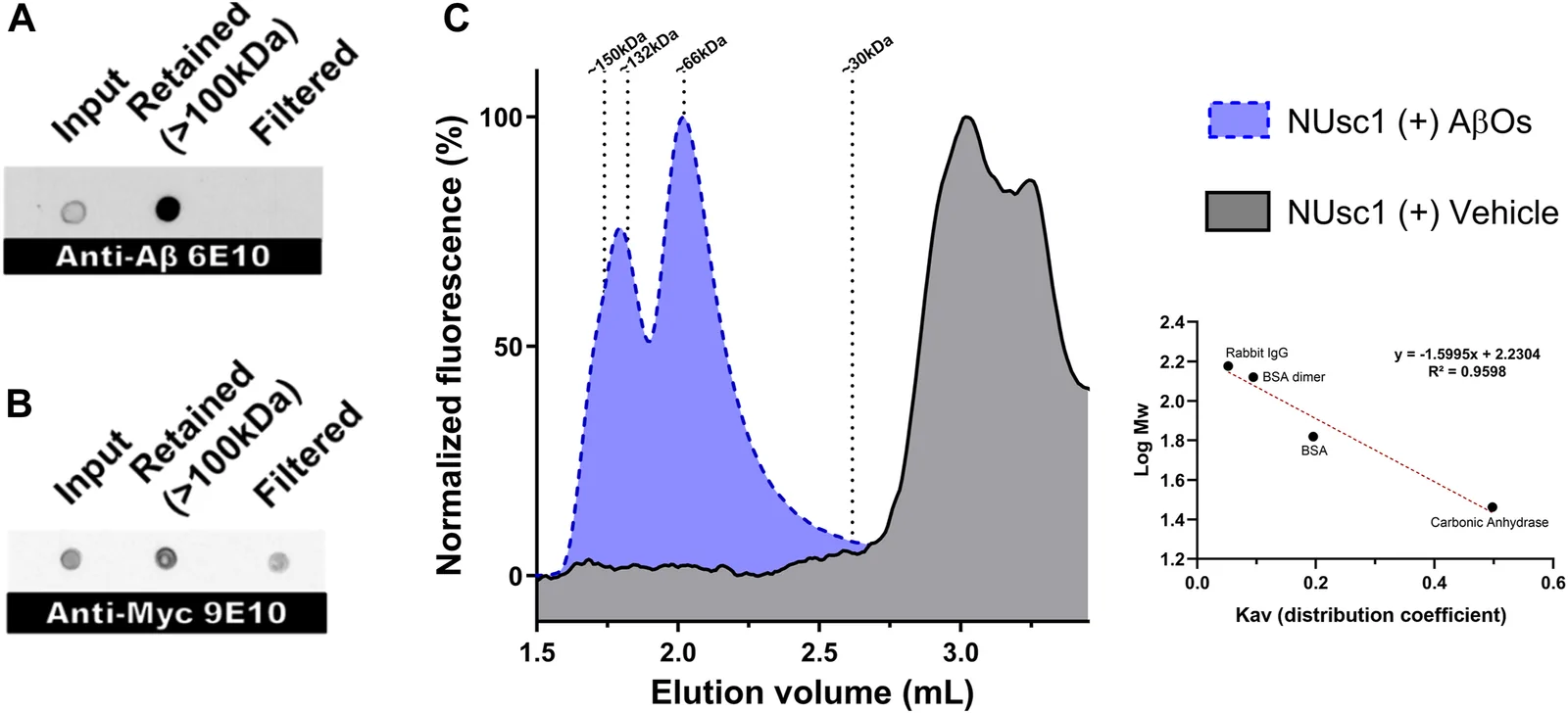
**Apparent molecular mass determination of the NUsc1–AβO Complex Using non-denaturing Filter-Based Fractionation and SEC.** AβOs were incubated with magnetic beads functionalized with scFv NUsc1. The eluted NUsc1–AβO complex was passed through a 100 kDa molecular weight cutoff filter. Dot blot analysis using (*A*) anti-Aβ 6E10 and (*B*) anti-c-myc 9E10 antibodies shows signal in the input (*left*), retentate (*center*), and filtrate (*right*) fractions. *C*, in addition, the apparent molecular mass of the complex was evaluated by size-exclusion chromatography (SEC). NUsc1-functionalized magnetic beads were incubated with vehicle (*gray*) or synthetic AβOs (*blue*), followed by elution with imidazole. Eluted proteins were analyzed by SEC on an HPLC system, with detection based on intrinsic tryptophan fluorescence (excitation: 295 nm; emission: 325 nm). Runs were performed in PBS (pH 7.2) at a flow rate of 1 ml/min. Apparent molecular mass was estimated by using a calibration curve plotting log (MW) against the distribution coefficient (Kav). Kav was calculated as (Ve − Vo)/(Vt − Vo), where Ve is the elution volume, Vo is the void volume, and Vt is the total column volume.

### Sizing of NUsc1-immunoprecipitated AβOs derived from 5xFAD mouse brain

Next, we employed the NUsc1-based immunoprecipitation assay to selectively pull down pathologically relevant AβO species from AD-transgenic mice brain. Dot blot results clearly showed that NUsc1 efficiently immunoprecipitates AβOs from 5xFAD brain homogenates (Fig. 7*A*). AβO signal was detected in all transgenic animals’ brain extracts tested, whereas age-matched wild-type extracts rendered no signal. On average, 0.4 fmol of AβOs were recovered per μg of total protein from transgenic brain homogenates (Fig. 7*B*). In addition, Western blot analysis indicated that the pulled-down species are mainly high molecular weight aggregates, based on the abundant signal above 250 kDa observed (Fig. S6). Next, brain-derived AβOs isolated from 5xFAD mouse brain using NUsc1-based immunoprecipitation followed by individual ion mass spectrometry (I^2^MS). I^2^MS analysis revealed a heterogeneous, multispecies distribution in transgenic samples, with distinct populations spanning up to ∼250 kDa (Fig. 7*C*), consistent with the higher–molecular weight species observed by Western blot analysis (Fig. S6). This distribution supports the presence of larger oligomeric assemblies. As observed with synthetic AβOs, the mass spectra displayed regularly spaced peaks, suggestive of stepwise oligomerization. Notably, species above ∼100 kDa appeared to increase in increments consistent with the addition of Aβ dimers or trimers, as indicated by shifts along the m/z axis. As with synthetic preparations, partial fragmentation during alkaline elution or electrospray ionization cannot be excluded. Overall, these results demonstrate that our NUsc1-based assay isolates AD-relevant AβO species from brain tissue, enabling selective capture and allowing a characterization of toxic oligomers under native-like conditions.

**Figure 7:**
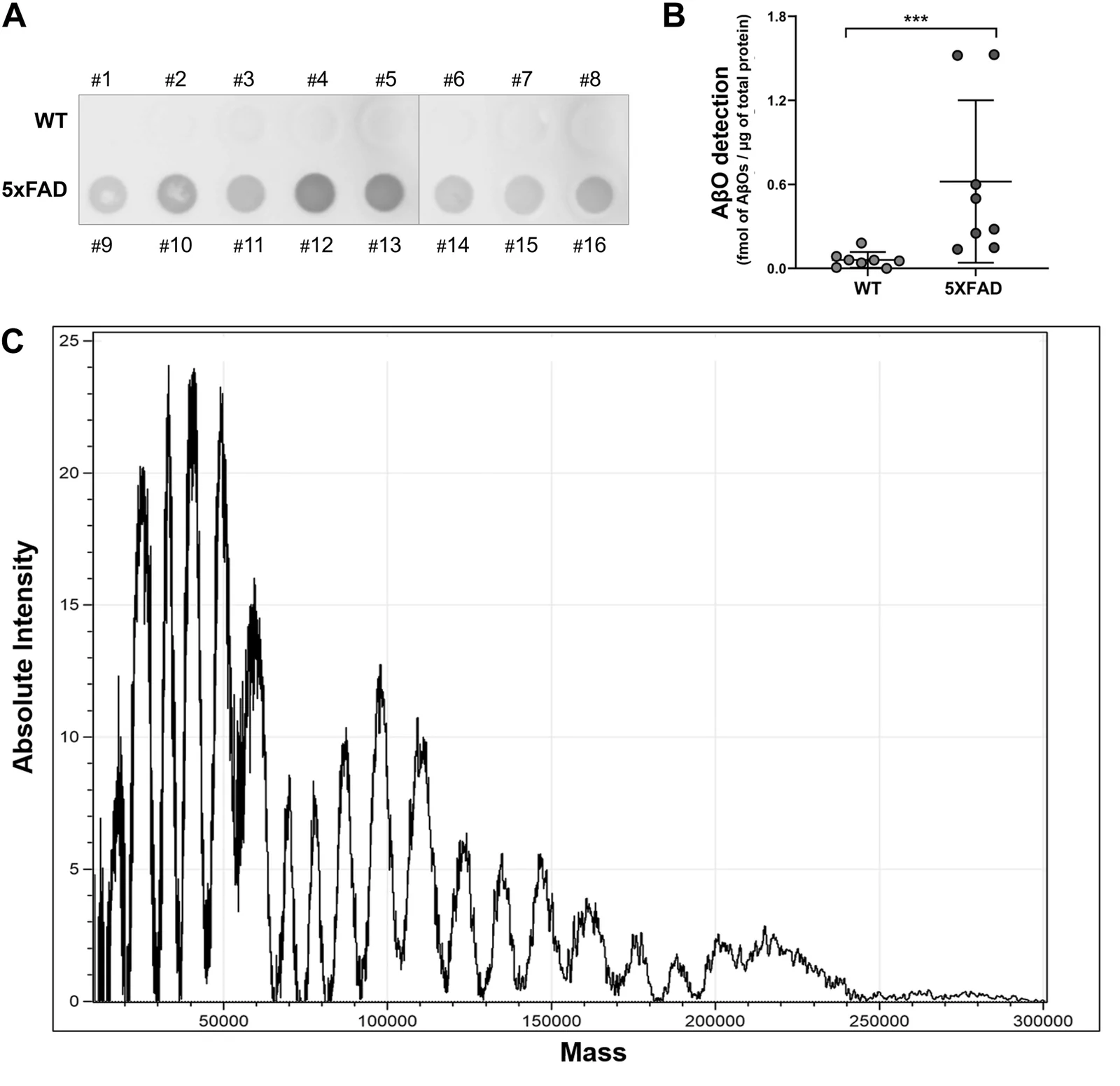
**Isolation and characterization of brain-derived AβOs immunoprecipitated from 5xFAD mouse brain extracts.***A*, dot blot of eluted samples from wild-type and transgenic mice brains. Homogenates from 8-months old animals (WT n = 8; 5xFAD n = 8; numbered as 1–16) were analyzed in two independent experiments. Membranes were probed with the α-AβO antibody NU2. *B*, AβO levels were quantified by densitometry using ImageJ, normalized by total protein input and plotted with GraphPad Prism. Semi-quantitative analysis was performed using a standard curve generated from synthetic AβOs run in parallel on the membrane (standard curve shown in Fig. S5). Statistical analysis was conducted using the Mann–Whitney test (*p* = 0.0006). *C*, individual ion mass spectrometry (I^2^MS) spectra in the mass domain showing native Aβ oligomers immunopurified from 5xFAD brain extracts using the NUsc1 antibody. The y-axis represents absolute intensity, reflecting oligomer abundance.

### Assessing the diagnostic potential of NUsc1 for selective detection of AβOs

Supported by evidence showing that NUsc1 can isolate and facilitate the characterization of toxic Aβ oligomers, and by prior work highlighting its therapeutic promise (9, 10, 12), this study further investigated an additional dimension of NUsc1: its potential application in the molecular diagnosis of AD. This is especially pertinent given the current diagnostic challenges, considering that non-invasive methods for early and definitive confirmation still remain scarce as well as the need for antibodies that specifically target and discriminate between soluble toxic species (30, 31, 32, 33).

We first assessed whether NUsc1 could bind to neuronal cells decorated with AβOs and ultimately developed a sensitive sandwich ELISA. Given that NUsc1 originates from a phage-display library and can therefore be produced in a phage-bound format, we used its phage-bound version (pbNUsc1) to evaluate its utility as a diagnostic tool. This approach is particularly attractive since it allows targeting the phage itself as a secondary target, rather than the scFv. The presence of multiple copies of phage coat proteins per virion provides multiple binding sites for the secondary peroxidase-conjugated antibody, resulting in signal amplification.

Immunofluorescence analysis revealed that pbNUsc1 partially detects synthetic fluorescently (FAM)-labeled AβOs bound to cultured neuronal cells (Fig. 8, *A*–*C*). Quantitative analysis showed an average of 30% colocalization between NUsc1 and AβO signals, with FAM-labeled AβOs contributing a higher overall signal intensity, indicating greater abundance compared to NUsc1-detected species. (Fig. 8*D*), suggesting selective binding of NUsc1 to a subset of AβO species, as also reported by Sebollela *et al.* (2017). This level of detection was consistent across two independent experiments. Colocalization was assessed using the Manders coefficient M1. Although pbNUsc1 does not recognize the entire AβO population, these results suggest that NUsc1 exhibits high sensitivity to specific protein oligomeric motifs, likely reflecting selective binding to AβO conformers while excluding other oligomeric structures present on the neuronal cell surface.

**Figure 8:**
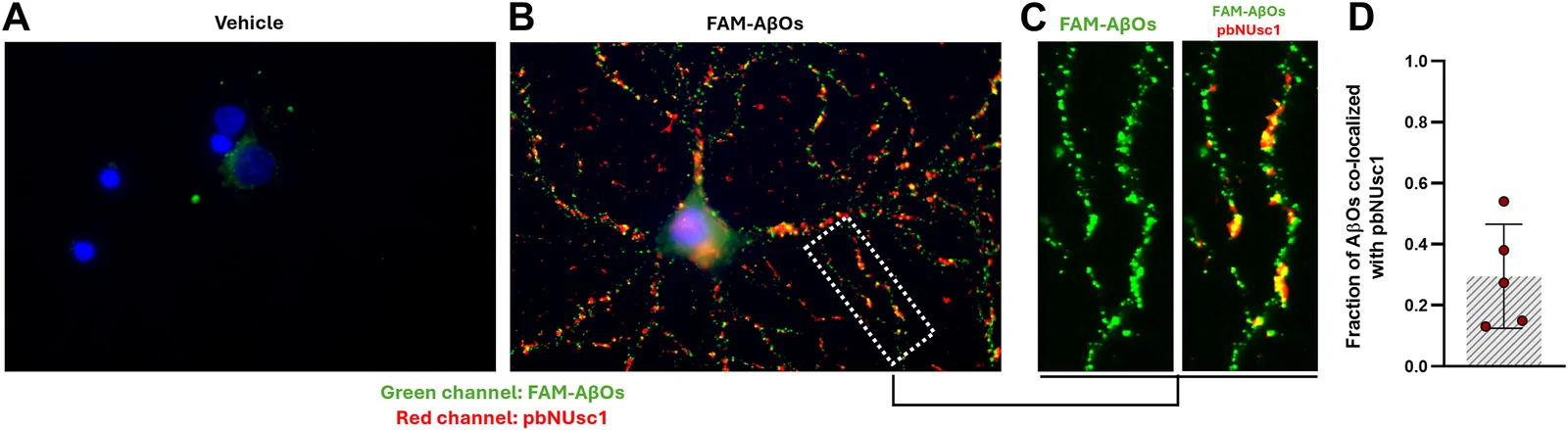
**NUsc1 detects specific AβO subpopulations in neuronal cultures.** Representative image of primary rat neuronal culture (DIV 18) exposed to (*A*) vehicle or (*B*) 250 nM fluorescent (FAM)-AβOs for 30 min. Co-localization between phage-bound NUsc1 and AβOs was assessed by directly comparing labeling for FAM-AβOs (*green*) with the pbNUsc1 signal (*red*). Nuclei were stained with DAPI (*blue*). *C*, Close-up visualization of pbNUsc1 and AβO distribution along neurites. *D*, quantification of the co-localization between FAM-AβOs and pbNUsc1 relative to the total FAM-AβOs signal (1.0).

In the sandwich ELISA, detection using pbNUsc1 was markedly more sensitive than the assay employing the commercial anti-AβIgG 22:35 (Fig. 9*A*). At certain concentration levels, the pbNUsc1-based assay yielded a saturated signal, whereas detection with 22:35 remained low and near the lower limit of its dynamic range. A measurable pbNUsc1-elicited signal above the blank was observed at AβO concentrations as low as approximately 12 pM (Fig. 9*B*), compared to 0.4 nM (Fig. 9*C*) for the 22:35 antibody-based assay, representing an approximate 32-fold increase in sensitivity. Notably, the signal achieved by the pbNUsc1 reaction required half the time of the control. This confirms that enhanced sensitivity is not due to longer reaction duration (Fig. 9, *A*–*C*). This result corroborates the notion that phage bound NUsc1 allows for significantly more secondary antibody binding events, thereby amplifying the detection signal. To validate the detection of AβOs in brain samples, oligomer abundance and size distribution were assessed in 5xFAD mouse brain extracts using the pbNUsc1 ELISA format (Fig. 9, *D* and *E*). Importantly, pbNUsc1 ELISA successfully detected AβOs in transgenic mouse brain extracts compared to age-matched wild-type controls (Fig. 9*D*). When the brain homogenates were fractionated by molecular size prior to ELISA, an interesting pattern emerged: pbNUsc1 signal was mainly associated with the molecular weight fraction larger than 30 kDa, and roughly equally distributed between 30-100 kDa and larger than 100 kDa (Fig. 9*E*). To further validate these findings in a physiologically relevant context, cerebrospinal fluid (CSF) collected from transgenic rats was analyzed using the pbNUsc1-based ELISA (Fig. 9*F*). Consistent with observations in 5xFAD mouse brain extracts, pbNUsc1 achieved reliable detection of AβOs in transgenic rat CSF across three independent replicates, clearly distinguishing transgenic from wild-type samples and highlighting its enhanced analytical sensitivity. Lastly and more strikingly, analysis of a small human CSF cohort using this ELISA (Fig. 9*G*) confirmed its potential to distinguish Alzheimer’s disease patients from controls, providing compelling evidence for the assay’s validity and translational potential in human samples. Sensitivity in these samples was enhanced using a luminescent substrate, reflecting the likely lower abundance of AβOs relative to Aβ42-overproducing transgenic models. Together, these findings demonstrate that pbNUsc1 preferentially binds higher-order (>30 kDa) soluble Aβ oligomers, consistent with previous reports (9) and highlight its strong potential as a sensitive tool for detecting distinct oligomeric subspecies in both accessible animal models and human biological fluids such as cerebrospinal fluid, reinforcing its translational applicability.

**Figure 9:**
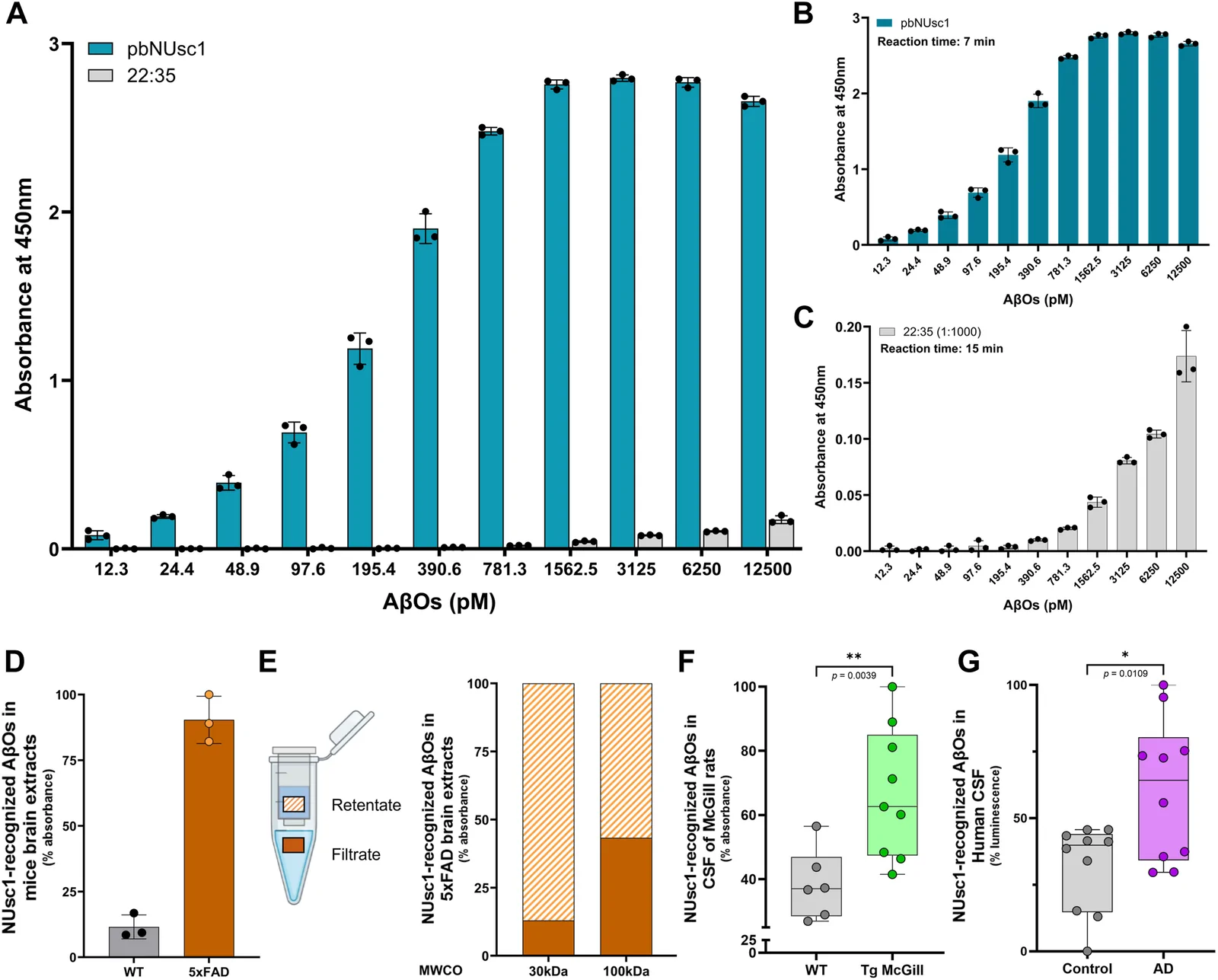
**pbNUsc1 ELISA enables detection of Aβ oligomers in synthetic, mouse brain–derived, and rodent and human CSF samples.***A*, sandwich ELISA detection comparing the signal obtained using phage-bound NUsc1 (pbNUsc1) or monoclonal anti-Abeta 22:35 IgG to detect synthetic AβOs. Isolated signals for (*B*) pbNUsc1 and (*C*) 22:35 detection are shown with adjusted signal axis scales to improve visualization and comparison. Reaction total times are shown for comparison. AβOs levels in brain homogenates from 15-month-old WT and 5xFAD mice were assessed by sandwich phage-bound NUsc1 ELISA. The signals obtained from non-fractionated samples of six animals are presented in (*D*), with absorbance at 450 nm normalized as a percentage of the highest measured value. Images from representative wells after signal development are shown at the top of the bars. Bars correspond to mean ± SD from six independent extracts. AβO levels in filtrates and retentates from a molecular weight based-centrifugal filtration using a 5xFAD brain extract as input were also assayed using the pbNUsc1-based ELISA (*E*). A scheme of the filtration device used, and corresponding MW fractions are depicted. *F*, CSF samples from WT (n = 6) and McGill-R-Thy1-APP (n = 9) rats, obtained from 8- to 12-month-old animals, were used. The data corresponds to the average of 3 independent experiments. ∗∗ *t* test with Welch’s correction, *p* = 0.0039. *G*, CSF samples from cognitively normal controls (N = 10) and Alzheimer’s disease (AD) participants (N = 10), matched for age and sex, were analyzed. The data shown corresponds to a representative result. ∗ *t* test with Welch’s correction, *p* = 0.0109. A curve of synthetic AβOs was used as a positive control in all ELISA plates, with a correlation coefficient R > 0.97, with absorbance at 450 nm or luminescence at 425 nm normalized as a percentage of the highest measured value.

## Discussion

Corroborating previous studies (9, 12), our present findings reinforce that NUsc1 is a promising candidate, not only for therapeutic development (10, 11), but as a valuable tool for advancing fundamental research in Alzheimer’s disease. In this paper, we demonstrate that NUsc1 exhibits nanomolar affinity for toxic Aβ oligomer sub-species, preventing their aggregation into fibrillar forms and allowing their selective isolation under mild conditions, a critical yet technically challenging step in Alzheimer's research. In addition, NUsc1 shows potential as a highly sensitive detection tool, surpassing conventional biochemical assays and offering a cutting-edge approach for identifying Aβ oligomers.

NUsc1 exhibits a noteworthy nanomolar-range affinity, comparable to that of clinically approved therapeutic antibodies targeting AβOs, including Aducanumab, Lecanemab, and Donanemab, all of which have received approval from the U.S. Food and Drug Administration (34, 35, 36, 37). Notably, its slow dissociation rate suggests prolonged target engagement, a property that may support enhanced clearance of Aβ oligomers. This is particularly advantageous for single-chain variable fragments (scFvs), which typically exhibit shorter *in vivo* half-lives than full-length IgG antibodies or Fc-fusion proteins, potentially extending the therapeutic window despite more rapid systemic clearance (38, 39). Furthermore, the high-affinity interaction of NUsc1 positions it as a valuable tool for selectively targeting and isolation of neurotoxic Aβ species. Such capacity is critical for enabling downstream studies aimed at characterizing these elusive oligomers and further elucidating their role in Alzheimer’s disease pathogenesis (40). While secondary interactions cannot be excluded at the highest concentrations tested, their contribution is clearly minor and unlikely to significantly impact the interpretation of the NUsc1–AβO primary interaction.

By interfering with Aβ aggregation pathways, NUsc1 may disrupt the secondary nucleation process associated with Aβ42 aggregation, a key driver of Alzheimer's disease progression (41, 42, 43, 44), particularly considering that synthetic AβO preparations only contain a minor population of monomeric Aβ. This is of considerable significance since nucleation is crucial for sustaining the continuous conversion of AβOs into larger neurotoxic protofibrils (17, 45, 46, 47, 48, 49) as well as mature Aβ fibrils. Although these fibrils are less toxic than other Aβ species, they still contribute to brain inflammation and can serve as a reservoir for the retro-production of AβOs and other soluble Aβ species (50, 51). Preventing the formation of fibrillar species through NUsc1 binding may be more beneficial than using antibodies that target fibrils, as antibody-mediated degradation has been shown to enhance toxicity by promoting the formation of additional soluble toxic species (52, 53, 54). Although further investigation is needed to understand how NUsc1 specifically prevents secondary nucleation, its strong and selective binding ability offers new opportunities to isolate and study these species, helping to identify motifs and conformations associated with neurotoxicity.

NUsc1 has previously been shown to selectively target a subpopulation of neurotoxic AβOs (9, 12). The dose-dependent inhibition of AβO binding is consistent with NUsc1's selective interaction with oligomeric assemblies. Consistent with this finding, human neuronal-like cells exhibited diminished AβO-induced toxicity in the presence of NUsc1 (Fig. S2). With AβO levels held constant, it is reasonable to assume that low levels of NUsc1 mainly target high-affinity oligomers, while higher NUsc1 levels progressively bind lower affinity ones, eventually causing full inhibition. Consistent with previous and present findings demonstrating nanomolar affinity and specificity, these results position NUsc1 as an interesting choice for the specific isolation of disease-relevant AβO species under non-denaturing conditions prior to structural investigations.

Isolation of AβO species in their native conformation is particularly relevant given that most studies isolating AβOs for further characterization employ harsh acidic or detergent-based elution conditions, which can disrupt aggregated assemblies and predominantly result in the visualization of low-molecular-weight oligomers or monomers (55, 56, 57, 58, 59, 60). Moreover, isolating AβO species as part of a complex may enhance their stability, facilitating their analysis by biochemical and biophysical techniques. Recent advances in cryo-EM have enabled high-resolution visualization of intact protein complexes, surpassing earlier approaches that focused mainly on individual protein targets. Complexes incorporating scFv molecules may be particularly amenable to this technique due to the wealth of structural data already available for scFv fragments (61, 62, 63). As these methodologies converge, atomic-level insights into the architecture of neurotoxic oligomers may soon become attainable.

Knowing that NUsc1 preferentially targets AβOs larger than 50 kDa (9, 12), it is likely that the Western blot results presented here, ran in denaturing conditions, provide limited information regarding oligomer sizing. This interpretation is substantiated by several reports indicating that detergents such as SDS can alter the aggregation state of Aβ (64, 65, 66, 67). Inspired by previous work using the anti-AβO monoclonal antibody NU4 to indirectly estimate the size of AβOs (68), our findings using the scFv NUsc1 identified a NUsc1–AβO complex with apparent molecular weight of approximately 100 kDa. Based on the known size of NUsc1, this suggests that the predominant AβO species alone range from roughly 60 to 90 kDa, consistent with prior reports identifying high molecular weight AβOs as primary neurotoxic entities (2, 69, 70, 71, 72, 73, 74, 75), and in line with the notion that higher-order oligomers represent the majority of AβO species in Alzheimer’s disease brains (76). It is important to mention that alternative configurations beyond the assumed 1:1 stoichiometry with a monovalent ligand fragment cannot be excluded, including a 2:1 NUsc1:AβO ratio that may account for the larger species observed.

AFM analysis provides important insight into the physical characteristics of the species preferentially bound by NUsc1 and suggests a relatively narrow distribution compared to the broader heterogeneity typically observed in synthetic AβO preparations (77, 78, 79, 80, 81, 82). A comparison with previously published data on synthetic AβOs (77) supports the interpretation that the species detected in this study represent only a subset of the total population generated during *in vitro* aggregation, as has been observed for other isolated species using different anti-AβO antibodies (68). This observation reinforces the idea that NUsc1 selectively targets a distinct conformational subset of AβOs, potentially associated with neurotoxicity.

I^2^MS analysis of synthetic AβOs targeted by NUsc1 revealed a population dominated by fragmented, low-molecular-weight species, with a notably higher abundance of trimers and tetramers, although oligomers up to ∼50 kDa were also detected. Meanwhile, I^2^MS analysis of NUsc1-isolated AβOs from 5xFAD mouse brain revealed a wider molecular weight distribution, with defined populations up to ∼250 kDa. This larger size likely reflects the complex environment in which brain-derived AβOs are generated. Unlike synthetic oligomers, *in vivo* Aβ monomers may undergo post-translational modifications and exhibit distinct aggregation patterns. Moreover, brain-derived AβOs typically require lower amounts to induce toxicity *in vitro* and *in vivo*, raising possible slight conformational differences between synthetic and brain-derived oligomers, and highlighting their pathological relevance (83). Additionally, these findings likely reflect, at least in part, the influence of both the immunoprecipitation protocol optimized for mass spectrometry, which involved the use of epoxy beads and alkaline elution, and the ionization process, which may result in sample fragmentation. This may account for the prevalence of smaller oligomeric species. Actually, the predominance of small oligomers under these somewhat denaturing conditions is consistent with the notion that these were disassembled from higher-order aggregates, although the detected trimers and tetramers may still represent biologically relevant intermediates within the Aβ aggregation pathway (84, 85, 86, 87, 88), indicating that a low-order organizational motif may contribute to the formation of these pathogenic larger assemblies.

In addition to its value for basic research and therapeutic development, NUsc1 may also hold potential as a diagnostic tool, particularly in light of the current lack of an accessible molecular diagnostic to support early diagnosis of Alzheimer's disease (89, 90, 91). Current molecular diagnostic techniques for confirming Alzheimer's disease, such as PET imaging or single-molecule enzymatic detection, are often costly and not widely accessible, especially given the global prevalence of the disease and its expected rise in the coming decades. Moreover, these methods frequently exhibit limited specificity for neurotoxic entities, primarily targeting soluble Aβ40/Aβ42 peptides rather than aggregated species, lacking sensitivity for low concentrations of soluble toxic oligomers, especially in CSF or blood samples (92, 93, 94, 95, 96, 97). NUsc1 ability to selectively detect AβOs harboring primary neurons suggested that it could be adapted for use in other techniques or quantitative assays like ELISA, for sensitive detection of these oligomeric species in tissue extracts, or even peripheral samples from human subjects, to assist in molecular diagnostic confirmation or early detection, as AβO accumulation begins decades before clinical symptoms manifest (48, 98, 99).

Our ELISA results clearly demonstrate that pbNUsc1 exhibits higher detection sensitivity compared to a commercial anti-Aβ antibody, with an approximate 32-fold increase in sensitivity. This enhanced performance is likely attributed to the integration of an anti-AβO conformational capture antibody with the use of NUsc1 displayed on a phage particle. Since the phage coat protein is present in thousands of copies per phage (100), each NUsc1 molecule bound to antigen benefits from substantial signal amplification. Moreover, the use of conformational antibodies, rather than pan–anti-Aβ antibodies that recognize linear epitopes regardless of protein structure, resulted in increased signal intensity (Fig. S7*A*). This improvement likely reflects the fact that many species detected by linear epitope–recognizing antibodies are not efficiently recognized by NUsc1, whereas anti-AβO antibodies preferentially capture conformations compatible with NUsc1 binding. Among the antibodies tested, NU1 exhibited the most robust and consistent detection when paired with phage-bound NUsc1 (Fig. S7*B*). Remarkably, pbNUsc1 could detect brain-derived AβOs across multiple AD models and human samples, including a limited human CSF cohort of AD patients. These findings support the potential of this assay as a future tool for molecular diagnosis in human CSF or even plasma, in which detection of specific neurotoxic Aβ oligomer subsets remains challenging. Although further comprehensive and rigorous studies are required to establish its diagnostic value, these early results provide a solid foundation for future studies. Importantly, this work represents a meaningful step forward in the development of tools aimed not just at detecting Aβ in general, but specifically the toxic oligomeric forms implicated in neurodegeneration, which may be present years before senile plaques are formed.

In summary, our findings establish NUsc1 as a valuable tool in characterizing the structural nature of Aβ oligomers, offering important insights into their role in Alzheimer's disease pathology. NUsc1 also shows promise for the development of highly specific and sensitive diagnostic methods, representing a significant advancement toward early or even pre-symptomatic detection of AD. Together, our data support the use of NUsc1 to advance scientific research on Alzheimer's disease and facilitate clinical development of more effective diagnostic strategies.

## Experimental procedures

### Preparation of synthetic Aβ oligomers

AβOs were prepared as in (77). Shortly, Aβ (1–42) peptide (Echelon Biosciences) film was brought to room temperature and dissolved to 5 mM using dimethyl sulfoxide. Ham’s F12 medium buffer was added to make the final peptide concentration to ∼100 μM. The mixture was then vortexed for ∼10 s and incubated overnight at 4 °C. The next day, the mixture was centrifuged at 14,000*g* for 10 min at 4 °C. The supernatant (oligomeric product) was quantified using a Bradford assay, and the concentration was calculated based on the monomeric molecular weight of Aβ42. The AβO preparation was then aliquoted for single-time use and stored at −80 °C. Alternatively, Aβ (1–42) films were dissolved in dimethyl sulfoxide and diluted into sodium borate buffer (25 mM borax, pH 8.5), followed by cross-linking with 1,5-difluoro-2,4-dinitrobenzene (DFDNB) to generate stable oligomeric preparations for cell-based incubation at 37 °C, as previously described in Cline *et al.*, 2019 (21).

### NUsc1 production and purification

The anti-Aβ oligomer (AβO) single-chain variable fragment (scFv) NUsc1 was selected from the Tomlinson I + J human scFv phage display library through panning, following the procedure described in Sebollela *et al.* (9). Consistent with scFvs from this library, NUsc1 contains His and Myc tags (Fig. S8). NUsc1 was expressed in *Escherichia coli* HB2151 cells induced with isopropyl β-D-1-thiogalactopyranoside (IPTG), essentially as outlined in Barbas III *et al.* (100). Secreted NUsc1 was recovered from the culture supernatant and purified using a Protein A affinity column, as described by Nissim *et al.* (101). Non-secreted NUsc1 was extracted from the cell pellet *via* chemical and mechanical lysis, followed by purification using a nickel-nitrilotriacetic acid (Ni-NTA) affinity column and size-exclusion chromatography (SEC) on a HiLoad 16/600 Superdex 75 pg column (Fig. S1). Protein concentration was determined using the Bradford assay (102). Purified NUsc1 was stored in 1 × phosphate-buffered saline (PBS), pH 7.4, at −80 °C until further use. Phage-bound NUsc1 (pbNUsc1) was generated following procedures adapted from Barbas (100) and Velasco *et al.* (12). TG1 cells were infected with KM13 helper phage and cultured overnight in 2xTY medium with ampicillin and kanamycin. After removing bacterial debris by centrifugation, the phage-rich supernatant was purified through two PEG/NaCl precipitation steps. The purified phage was resuspended in PBS, supplemented with 15% glycerol, and stored at −80 °C.

### Mouse model

Analysis of 8- and 15-month-old *Mus musculus* wild-type and 5xFAD transgenic mice (103) was approved by the Institutional Animal Care and Use Committee at Northwestern University under the protocol #IS00004010. Mice were euthanized by cervical dislocation, and whole mouse brain tissue was harvested and flash-frozen prior to extract preparation.

### Tissue homogenization

Brain tissue was homogenized in phenol red-free Ham’s F-12 medium (Thermo Fisher, Cat. #11550043) supplemented with Halt Protease Inhibitor Cocktail (Thermo Fisher, Cat. #78438) at a ratio of 1 ml buffer per 100 mg of tissue. Tissue samples were weighed and homogenized on ice using a handheld homogenizer for three 10-s cycles. The resulting homogenate was centrifuged at 14,000*g* for 20 min at 4 °C. The supernatant was carefully collected into low-protein-binding microcentrifuge tubes. Total protein concentration was determined using the Bradford assay (102). Homogenates were aliquoted and stored at −80 °C until further use.

### Rat model

The transgenic rats used in this study belonged to the McGill-R-Thy1-APP line, which expresses the human APP751 transgene carrying two familial Alzheimer’s disease mutations—Swedish (K670N/M671L) and Indiana (V717F)—under the control of the murine Thy1.2 promoter (104, 105). Experimental animals were 8 to 12 months old and included heterozygous transgenic rats (n = 9) and their non-transgenic wild-type littermates (n = 6). All experiments were conducted in accordance with the Institutional Committee for Care and Use of Laboratory Animals (CICUAL) of the School of Medicine, University of Buenos Aires (Argentina), and in compliance with the *Guide to the Care and Use of Experimental Animals* of the Canadian Council on Animal Care (CCAC, Canada), as well as the *Guide for the Care and Use of Laboratory Animals* from the Office of Laboratory Animal Welfare, National Institutes of Health (NIH, USA).

### Collection of cerebrospinal fluid from rats

Prior to CSF collection, animals were anesthetized with a ketamine/xylazine mixture (1:1) and positioned in a stereotaxic frame. Fur over the neck was trimmed, the area was rinsed with distilled water, and then disinfected with 70% ethanol. The rat’s head was flexed downward at ∼45°, exposing the rhomboid-shaped surface between the occipital protuberances and the atlas. CSF was collected from the cisterna magna following Nigori *et al.* (106). A 23-G needle was inserted without incision and connected *via* 10-inch PE-50 tubing to a collection syringe. Clear CSF was slowly aspirated while monitoring for blood contamination; if blood appeared, the tubing was pinched and cut above the contamination point before continuing collection. Typically, 100 to 150 μl of CSF was obtained per animal. Body temperature was maintained with a heating pad. The procedure lasted approximately 10 to 15 min per animal, after which rats were placed in a heated recovery chamber until fully awake.

### Human participants

All individuals provided informed consent for protocols approved by the Emory Institutional Review Board. Participants included volunteers in the Emory Goizueta Alzheimer’s Disease Research Center (GDRC; IRB00079069), Emory Healthy Brain Study (EHBS; IRB00080300) (107), and patients seen in the GADRC-affiliated Cognitive Neurology Clinic (IRB00078273). All participants were diagnosed by expert GADRC UCNS certified Cognitive and Behavioral Neurologists based on history, examination, detailed cognitive testing, neuroimaging, and CSF biomarker testing (Tables 1, S2 and S3). All human studies were conducted in accordance with the ethical standards of the institutional research committee and with the principles of the Declaration of Helsinki.

**Table 1:** Demographic, cognitive, genetic, and biomarker characteristics of control and Alzheimer’s disease participants

Characteristics	Control (N = 10)	AD (N = 10)	*p* value
Age	70.2 ± 6.9	70.2 ± 7.2	0.995
Female	5 (50%)	5 (50%)	>0.9999
Education	17.1 ± 2.3	15.6 ± 2.5	0.175
MoCA	27.0 ± 3.2	19.2 ± 3.3	<0.0001
APOE e4+	0 (0%)	6 (60%)	0.003
Aβ42 (pg/ml)	1755 ± 565	606 ± 186	<0.0001
tTau (pg/ml)	187.9 ± 43.5	356.0 ± 173.3	0.014
pTau (pg/ml)	16.0 ± 4.4	36.4 ± 18.0	0.006

Participants were matched for sex and age, and all participants were White. AD, Alzheimer’s disease; APOE ε4+, at least one ε4 allele; Aβ42, amyloid-β 1 to 42; MoCA, Montreal Cognitive Assessment; pTau, phosphorylated tau; tTau, total tau.

### Collection of cerebrospinal fluid and analysis

Participants were asked to fast prior to visits, and most procedures were performed before noon. Lumbar punctures were done using a 24 g atraumatic Sprotte spinal needle (Pajunk Medical Systems, Norcross, GA) with aspiration, transferred to 15 ml polypropylene tubes (Corning, Glendale, AZ), aliquoted into 0.5 ml volume in 0.9 ml FluidX tubes (Azenta, Chemsford, MA), and placed into dry ice/methanol bath prior to transfer to −80 °C freezers. Time from collection to freezing was less than 60 min. CSF Aβ_42_, tTau, and pTau assays were performed following a single freeze-thaw on a Roche Cobas e601 analyzer using the Elecsys Gen2 platform (108).

### Biolayer interferometry (BLI)

The single-chain variable fragment (scFv) NUsc1 was biotinylated using the EZ-Link NHS-PEG4 Biotinylation Kit (Thermo Scientific), following the manufacturer’s instructions. Biotinylated NUsc1 was immobilized onto High Precision Streptavidin (SAX) biosensors (Sartorius). Prior to loading, SAX biosensors were equilibrated in phosphate-buffered saline (PBS). Immobilization was performed by incubating the sensors with a 200 nM NUsc1 solution in PBS, followed by a washing step in PBS to remove unbound material. Following immobilization, the NUsc1-functionalized sensors were used for affinity measurements against synthetic Aβ oligomers prepared/diluted in F12 buffer, which includes free biotin to serve as a blocking agent. Analyte concentrations ranged from 31.25 nM to 500 nM, and binding interactions were assessed using a standard association-dissociation protocol: 900 s for the association phase and 900 s for the dissociation phase. Real-time changes in optical thickness due to molecular interactions were recorded using the Octet BLI Discovery System (Sartorius). Binding kinetics and affinity constants were derived from the sensorgrams using the Octet BLI Discovery Software, applying a 1:1 Langmuir binding model for global data fitting, where Rmax was allowed to vary across analyte concentrations.

### Hippocampal neuron cell culture

Fresh embryonic day 18 (E18) Sprague-Dawley rat hippocampus, cortex, and subventricular zone tissue was obtained as a combined sample from Transnetyx and cultured according to the manufacturer’s protocol, based on the procedures described by Brewer *et al.* (109, 110). Cells were dissociated, diluted to a final concentration of 0.11 to 0.22 × 10^6^ cells/ml, and plated at 0.15 ml per coverslip onto sterile 12 mm circular coverslips pre-coated with poly-D-lysine. Coverslips were arranged four per 35 mm culture dish, resulting in a plating density of approximately 15,000 cells/cm^2^. Cultures were fed on day 4 post-plating by replacing half of the medium with fresh NbActiv1 and subsequently maintained with half-media exchanges every 2 to 3 days using NbActiv1.

### AβO treatment of mature hippocampal neurons

Mature hippocampal neurons at 24 days *in vitro* (DIV) were treated with 500 nM Aβ oligomers (AβOs) that had been pre-incubated with varying concentrations of the NUsc1 antibody (50–1000 nM) in 1 × PBS. The AβO-antibody mixtures were incubated for 1 h at 37 °C prior to application. Neuronal cultures were then treated with the pre-incubated complexes for 1 h at 37 °C. For immunofluorescence analysis, cells were washed with sterile PBS and fixed with formaldehyde.

### Immunofluorescence staining and image analysis

Following treatment, coverslips were washed with sterile 1 × PBS and fixed by the sequential addition of 3.7% formaldehyde: first, by adding an equal volume of 3.7% formaldehyde solution for 10 min, followed by complete replacement with fresh 3.7% formaldehyde for an additional 10 min at room temperature. Fixed cells were then washed three times with PBS and blocked overnight at 4 °C in a blocking solution containing 5% normal goat serum and 0.1% Triton X-100 in PBS. Primary antibodies—NU2 (1–2 μg/ml, in-house) and CPCA-MAP2 (1:10,000)—were diluted in 10% blocking buffer and incubated with the samples overnight at 4 °C. The next day, coverslips were washed three times with PBS and incubated for 3 h at room temperature in the dark with secondary antibodies diluted in 10% blocking buffer: goat anti-mouse Alexa Fluor 488 (1:2000) and goat anti-chicken Alexa Fluor 568 (1:2000). After three final PBS washes, coverslips were mounted using ProLong Diamond Antifade Mountant with DAPI. Fluorescence images were acquired using a Leica DM6B fluorescence microscope at 63 × magnification. Image stacks were processed using Leica LAS-X software with 3D deconvolution (three iterations) followed by maximum intensity projection. Quantification of AβO puncta per micron of dendrite length was performed on 10 images per condition using ImageJ. TIFF images were randomized before analysis to minimize bias, and neuronal cell bodies were manually traced and removed to exclude nonspecific antibody accumulation. Fluorescence channels were then separated by wavelength. Total dendrite length was quantified from the MAP2 signal by applying a Gaussian blur, thresholding each image uniformly, skeletonizing the resulting signal, and measuring total skeleton length. To quantify AβO puncta along dendritic processes, the MAP2 signal was similarly blurred and thresholder, then expanded by 15 dilations to include nearby synapses and fine dendritic projections that extended beyond regions of strong MAP2 fluorescence. Within this selected area, NU2-positive puncta above a uniform threshold were quantified using ImageJ particle analysis. Threshold values were determined using positive and negative control images. Final AβO puncta density was calculated by dividing total NU2 puncta by total dendrite length. Dose-response curves were generated in GraphPad Prism using nonlinear regression with a sigmoidal fit and Hill slope. The calculated IC_50_ value was 0.1 μM (R^2^ = 0.73).

### NUsc1 inhibition of Aβ fibril formation

A Thioflavin T (ThT) stock solution was prepared as described by Krebs *et al.* (14) by dissolving ThT at a stock concentration of 40 μM in saline phosphate buffer (pH 7.2). The stock solution was diluted to 10 μM in PBS and combined with AβOs (10 μM) and/or NUsc1 (4.75 μM) to generate four experimental conditions: (1) NUsc1, (2) NUsc1 + AβOs, (3) AβOs, and (4) Aβ fibrils. Duplicate samples of each condition were loaded into a 96-well black-bottom microplate and maintained on ice prior to incubation. The plate was incubated at 37 °C on a shaker set to 50 rpm. Fluorescence measurements were recorded at time zero and every four hours for 24 h using an excitation wavelength of 482 nm and an emission wavelength of 450 nm. Following the fluorescence assay, samples were analyzed by transmission electron microscopy (TEM) to assess fibril morphology.

### Transmission electron microscopy (TEM)

For TEM analysis, 300-mesh copper grids were floated on 20 μl droplets of each sample condition and incubated for 2 min. Grids were then washed twice with double-distilled water (ddH_2_O) for 10 s each and immediately stained with 0.75% uranyl acetate for 20 s. After staining, grids were air-dried for 30 min at room temperature. Imaging was performed using a JEOL 1400 Flash transmission electron microscope equipped with a LaB_6_ filament and a Gatan OneView camera, operating at accelerating voltages ranging from 120 kV to 40 kV. Fibril thickness was quantified using ImageJ by first setting the scale based on the image’s scale bar. For each of six individual fibrils, four perpendicular measurements were taken along their length using the straight-line tool, and the mean thickness per fibril was calculated to assess overall fibril morphology.

### NUsc1-assisted isolation of AβOs under native condition

Magnetic bead-based immunoprecipitation was performed using cobalt-coated magnetic beads (Invitrogen) according to the manufacturer’s protocol with minor modifications. Beads were first vortexed for 30 s to ensure a homogeneous suspension. For each condition, 5 μl of bead suspension (equivalent to 0.2 mg) was incubated with 700 μl of NUsc1 antibody (14 μg/ml) diluted in binding/wash buffer, for 30 min at 4 °C under continuous end-to-end rotation. Beads were then separated using a magnetic stand and washed three times with 1 ml of binding/wash buffer. To prevent non-specific binding, NUsc1-coated beads were incubated with 700 μl of 0.75% BSA solution diluted in binding/wash buffer for 1 h at 4 °C. After blocking, beads were washed once with 1 ml of binding/wash buffer and incubated with 700 μl of synthetic AβO solution, at 0.8 μM, or alternatively with 2.1 mg of total protein mice brain homogenates, diluted in immunoprecipitation buffer for 2 h at 4 °C. Following incubation, beads were washed and eluted with 50 to 100 μl of elution buffer containing 400 μM imidazole on ice for 15 min without agitation. Eluates were concentrated using a 100 kDa molecular weight cutoff centrifugal filter and subsequently analyzed by Western and dot blot.

### Size-exclusion chromatography of the NUsc1–AβO complex

Eluates obtained from immunoprecipitation with NUsc1-functionalized beads incubated with either vehicle (control) or synthetic AβOs were analyzed. The eluate derived from AβO samples was further subjected to centrifugal filtration using 100 kDa cutoff membranes (Millipore, #UFC510024) at 10,000*g* for 10 min at 4 °C to remove unbound NUsc1. Samples were analyzed under non-denaturing conditions by size-exclusion chromatography on an Eprogen GPC-101 to 25 column (5 μm particle size, 100 Å pore size, 4.6 × 250 mm) connected to a Shimadzu HPLC system equipped with a fluorescence detector. Eluates (20 μl) were injected at a flow rate of 1 ml/min in 1 × PBS, pH 7.2. Detection was based on the intrinsic fluorescence of tryptophan residues in NUsc1 (excitation at 295 nm and emission at 325 nm). Plasmid DNA (pGEM-T) and tryptophan (Sigma, #T3300) were used to determine the void volume (Vo) and total column volume (Vt), respectively. The column was calibrated using carbonic anhydrase (29 kDa; Sigma, #C7025), bovine serum albumin (66 kDa; Sigma, #A7906), and non-specific rabbit IgG (150 kDa; Sigma, #I5006). A calibration curve was generated by plotting log(molecular weight) *versus* the distribution coefficient (Kav), calculated as Kav = (Ve − Vo)/(Vt − Vo), where Ve is the elution volume. Apparent molecular masses were estimated by interpolation from this calibration.

### Bead-based enzyme-linked immunosorbent assay (ELISA)

As an alternative to imidazole-based elution, NUsc1-functionalized magnetic beads bound to synthetic AβO species were subjected directly to a bead-based enzyme-linked immunosorbent assay (ELISA). Beads containing NUsc1/AβO complexes were incubated with 700 μl of the monoclonal anti-Aβ antibody 6E10 (1:2000; BioLegend) diluted in binding/wash buffer supplemented with 0.5% BSA, for 1 h at 4 °C. Following incubation, beads were washed three times with 1 ml of binding/wash buffer and incubated with 700 μl of HRP-conjugated anti-mouse IgG (1:20,000; BioLegend) in binding/wash buffer containing 0.5% BSA, for 1 h at 4 °C. After secondary antibody incubation, beads were reacted with 100 μl of 1-Step Turbo TMB substrate (Thermo Scientific) for 5 to 15 min in the dark. The enzymatic reaction was stopped by adding 100 μl of 2 M H_2_SO_4_. Beads were separated using a magnetic stand, and the resulting supernatant was transferred to a 96-well microplate. Absorbance was measured in a plate reader at 450 nm. An scFv control (kindly donated by Prof. Jose Elpidio Barbosa), functionalized onto magnetic beads using the same procedure, was included in parallel to assess the specificity of AβO binding and detection relative to NUsc1.

### Dot blot analysis of isolated NUsc1/AβOs complex

For dot blot analysis, NUsc1/AβO complexes were subjected to molecular weight-based separation using 100 kDa molecular weight cutoff filters (synthetic only). Both the retentate and filtrate fractions, along with non-centrifuged control samples and brain-derived samples, were diluted to a final volume of 200 μl and applied to a 0.45 μm nitrocellulose membrane, pre-equilibrated in 1 × PBS. Sample application was performed using a 96-well hybridization dot blot apparatus (Whatman). Membranes were air-dried at room temperature for 10 min and then blocked for 1 h at room temperature in 5% non-fat dry milk (NFDM) prepared in 1 × TBST. Following a brief wash in 1 × TBST, membranes were incubated with the conformational anti-AβO antibody NU2 (1 μg/ml) diluted in blocking solution, followed by incubation with HRP-conjugated anti-mouse secondary antibody at 1:20,000 dilution in the same blocking buffer. Immunoreactivity was detected using the SuperSignal West Femto chemiluminescent substrate (Thermo Scientific), and membranes were imaged using the Sapphire imaging system (Azure Biosystems).

### Western blot analysis of isolated NUsc1/AβOs complex

Protein samples were prepared by mixing eluates with 4 × Laemmli sample buffer (Bio-Rad) and resolved on 4 to 20% gradient Tris-Glycine gels (Novex, Thermo Fisher) alongside a Kaleidoscope molecular weight standard ladder (Bio-Rad). Electrophoresis was conducted in 1 × Tris-Glycine running buffer (Bio-Rad) at a constant voltage of 225 V for 35 min. Following electrophoresis, proteins were transferred to 0.45 μm nitrocellulose membranes (GE Healthcare Life Sciences) using a wet transfer system (Trans-Blot Cell, Bio-Rad) at 100 V for 1 h at 4 °C. Membranes were blocked in 5% non-fat dry milk (NFDM) prepared in 1 × TBST for 1 h at room temperature. Blocked membranes were incubated with the conformational anti-AβO antibody NU2 (1 μg/ml) in blocking solution, followed by incubation with HRP-conjugated anti-mouse IgG secondary antibody (BioLegend) at a 1:20,000 dilution. Chemiluminescent detection was performed using SuperSignal West Femto substrate (Thermo Scientific), and membranes were imaged using the Sapphire imaging system (Azure Biosystems).

### Atomic force microscopy analysis of NUsc1/AβO complex

Atomic force microscopy (AFM) was performed using a Bruker Dimension ICON system (Bruker, Billerica, MA) operating in Tapping Mode in air. TESPA-V2 probes (Bruker) with a nominal resonance frequency of ∼320 kHz, spring constant of ∼37 N/m, and tip radius of ∼7 nm. Sample preparation involved adsorbing 100 μl of eluate onto freshly cleaved V1-grade muscovite mica slides (Electron Microscopy Sciences, Hatfield, PA), followed by incubation at 25 °C for 50 min. Slides were subsequently plunged three times in ultrapure water to remove residual salts and gently dried under N_2_ flow. AFM imaging was conducted with a scan size of 2 μm × 2 μm, at scan rates between 1 and 2 Hz. Images were acquired at a resolution of 256 samples per line, with an integral gain of 0.2, proportional gain of 0.26, and an amplitude setpoint between 18 and 25 nm. Acquired images represented three-dimensional topographic data rendered as two-dimensional color-coded height maps. The lateral (X, Y) coordinates corresponded to positions on the mica substrate, while the vertical (Z) axis indicated height relative to the slide surface. Image analysis and Z-height quantification were carried out using NanoScope Analysis software (Bruker). Raw images were subjected to first-order plane flattening to correct for background tilt and low-pass filtering to suppress high-frequency noise. Representative cross-sectional height profiles were extracted from AFM six individual molecules selected per condition, with lengths (150–200 nm) chosen to capture the full local environment surrounding each molecule. The vertical (height) scale was kept consistent across all profiles to enable direct comparison of molecular heights, whereas lateral dimensions were not directly compared due to varying cross-section lengths.

### Analysis of AβO species isolated using NUsc1by top-down mass spectrometry

Purification of synthetic AβOs was performed using magnetic beads conjugated with the NUsc1 antibody, by conjugating 25 μg of NUsc1 antibody to 5 mg of Dynabeads M-270 epoxy magnetic beads (Thermo Fisher Scientific) in 500 μl of SB buffer, following the manufacturer’s protocol. The conjugation reaction was carried out overnight at 37 °C with end-over-end rotation in a protein low Bind microcentrifuge tube (Eppendorf). Following conjugation, 50 μl of antibody-bound beads were transferred to a new tube and blocked in 0.1% bovine serum albumin (BSA) at 4 °C for 1 h with continuous rotation. The blocking solution was then removed, and the beads were washed once with Tris-buffered saline 1XTBS.For immunoprecipitation, 1 μM of synthetic AβOs and 1 mg of 5xFAD brain extract were added to the blocked beads. The mixture was incubated at 4 °C for 1 h with gentle rotation. After binding, the beads were washed three times with 1X TBS-T (0.05% Tween-20), followed by three washes with 1XTBS. The carrying tube was replaced after the second TBS wash to minimize nonspecific carryover. Bound AβOs were eluted by incubating the beads with 30 μl of 150 mM ammonium hydroxide (pH 9.0, diluted in water). The eluates were aliquoted and diluted 100- to 1000-fold in 30 mM ammonium hydroxide for direct infusion into a Q Exactive Ultra High Mass Range (UHMR) Orbitrap mass spectrometer (Thermo Fisher Scientific). Samples were introduced *via* a heated electrospray ionization (HESI) source using a syringe pump at a flow rate of 1 to 2 μl/min. The source voltage was adjusted between 2.3 and 3.5 kV. Mass spectra were acquired using Individual Ion Mass Spectrometry (I^2^MS) with a resolving power of 140,000 at m/z 200 (∼1 s transient), as previously described (111).

### pbNUsc1 Co-localization with AβOs by immunofluorescence

Mature rat hippocampal neurons at 18 days *in vitro* (DIV) were treated with 100 nM of FAM-labeled AβOs. Neuronal cultures were treated for 30 min at 37 °C. For immunofluorescence analysis, cells were washed with sterile 1XPBS and fixed with formaldehyde as mentioned before. Coverslips were first blocked with 5% normal goat serum (NGS) in 1XPBS for 45 min at room temperature. After blocking, coverslips were incubated in a 50% (v/v) phage-bound NUsc1 supernatant solution, prepared as in Sebollela *et al.* 2017, diluted in blocking buffer. Incubation was carried out for 2 h at RT or overnight at 4 °C. Coverslips were then washed three times with 1XPBS for 5 min each. Following pbNUsc1 incubation, coverslips were treated with monoclonal anti-M13 phage antibody (GE Healthcare) diluted at 1:250 in blocking buffer (10-fold diluted in PBS). Incubation was conducted for 2 h at RT, followed by three 5-min washes with PBS. Fluorescent secondary antibody (anti-mouse IgG) diluted 1:500 in 10-fold diluted blocking buffer was added, and coverslips were incubated for 1 h at RT. After incubation, coverslips were washed three times with PBS for 10 min each. Coverslips were mounted using ProLong mounting medium and allowed to dry flat for at least 24 h at RT. Samples were imaged immediately after mounting using a Nikon Eclipse TE2000-U inverted fluorescence microscope at 60X magnification, with image acquisition performed *via* MetaMorph software. Immunofluorescence images from two independent experiments were analyzed using five selected fields. Within each field, 3 to 5 neuronal branches were identified for quantification of co-localization between AβOs (FITC channel) and pbNUsc1 (Texas Red channel). Texas Red images were resized to match FITC resolution using bicubic interpolation. Background subtraction was performed with a rolling ball radius of 5 pixels, followed by binarization of both channels. A binary mask was generated by summing the binarized images. Co-localization was quantified using the Coloc 2 plugin in ImageJ, with default point spread function (PSF = 3 px), assigning FITC as Channel 1 and Texas Red as Channel 2. Assuming 100% labeling in the FITC channel, pbNUsc1 signal was expressed as a fractional percentage (0–1) and plotted using GraphPad Prism.

### Sandwich ELISA for sensitive detection of AβOs

Wells of a 96-well plate were coated with 80 μl of monoclonal anti-AβO antibody NU1 (1 μg/ml) diluted in 1 × Tris-buffered saline (TBS), sealed with plastic film, and incubated overnight at 4 °C without agitation. The next day, wells were washed three times with 240 μl of 1 × TBS, followed by blocking with 200 μl of 5% non-fat dry milk in TBS for 1 h at room temperature (RT). After blocking, wells were washed once with 300 μl of TBS. For antigen capture, wells were incubated overnight at 4 °C under constant agitation with either a synthetic AβO standard curve (0–62.5 fmol) along with vehicle control, or mice brain homogenates at 100 μg/ml, or rat CSF at 1 mg/ml. Alternatively, fractionated 5xFAD mouse brain homogenates were also used as input. The following day, wells were washed three times with 300 μl of 1 × TBS containing 0.1% Tween-20 (TBST) and incubated with 80 μl of pbNUsc1 (1.0–1.5 × 10^11^ pfu/ml in blocking buffer) for 1 h at RT with constant agitation. After washing three times with 300 μl of TBST, wells were incubated with the secondary detection antibodies. For pbNUsc1 detection, 80 μl of HRP-conjugated anti-M13 antibody (0.2 μg/ml; Sino Biological) was added. As a control, an anti-Aβ 22:35 antibody (1 μg/ml in blocking buffer) was used with the same AβO standard curve, followed by detection with HRP-conjugated anti-rabbit IgG (0.2 μg/ml). All secondary antibody incubations were conducted for 1 h at RT with agitation. After secondary antibody incubation, beads were reacted with 80 μl of 1-Step Turbo TMB substrate (Thermo Scientific) for 5 to 15 min in the dark. The enzymatic reaction was stopped by adding 40 μl of 2 M H_2_SO_4_. Absorbance was measured in a plate reader at 450 nm. For human CSF samples, the pbNUsc1 ELISA was adapted to a luminescent format using white opaque 96-well plates. The signal was developed using SuperSignal ELISA Femto substrate (Thermo Scientific), and luminescence was measured immediately after substrate addition at 425 nm.

## Data availability

All data described are fully contained within this manuscript and its Supplementary Information files.

## Supporting information

This article contains supporting information.

## Conflict of interest

The authors declare that they have no conflicts of interest with the contents of this article.

## Figures and Tables

**Fig. S1:**
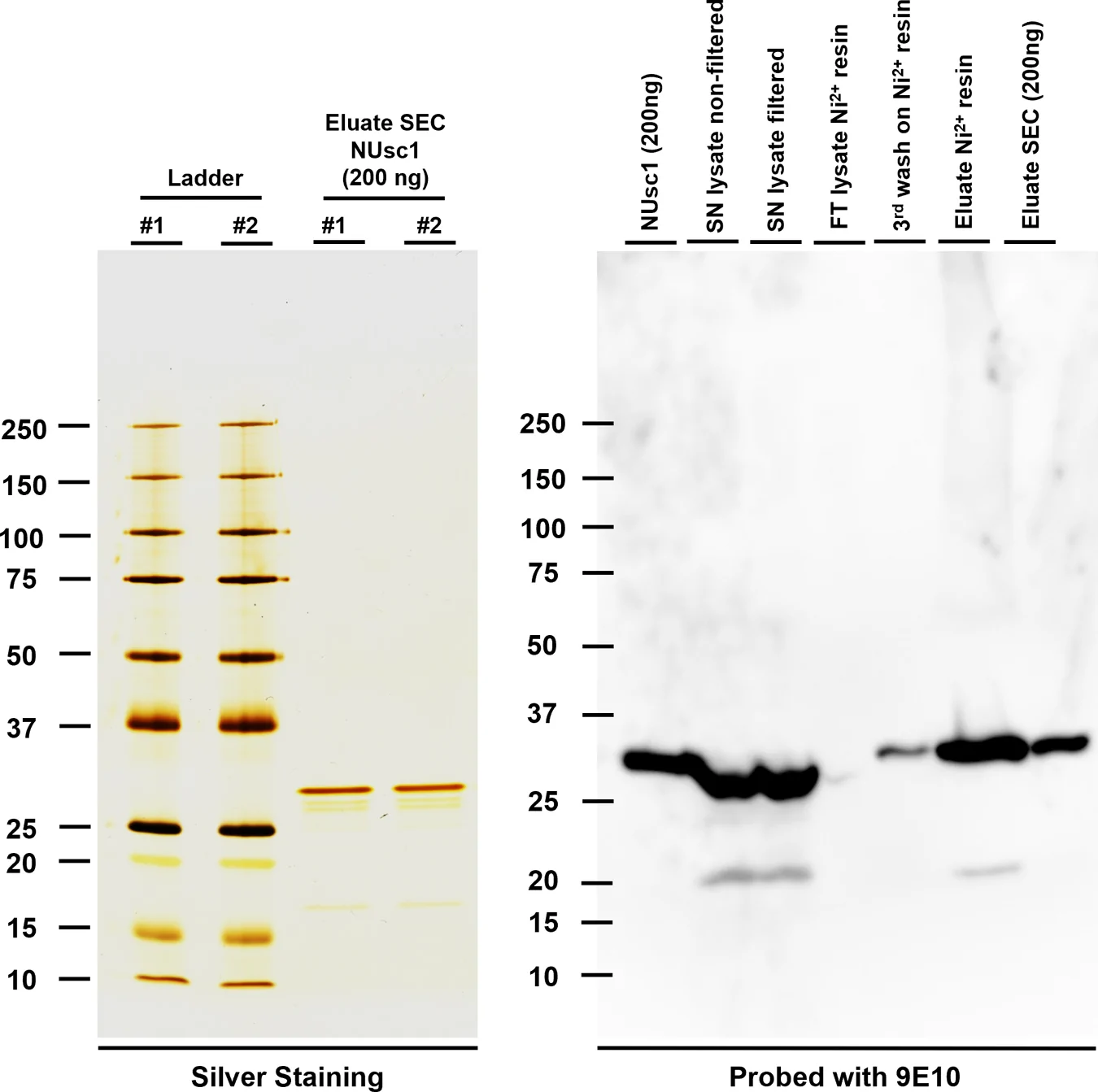
**NUsc1 production and purification quality control.** Representative silver-stained SDS-PAGE gel and western blot membrane showing NUsc1 expression and purification from E. coli HB2151. Samples include cell lysate supernatant, flow-through from Ni²⁺ resin, third wash from Ni²⁺ resin, Ni²⁺ resin eluate, and size-exclusion chromatography (SEC) eluate. NUsc1 corresponds to a protein of approximately 29 kDa. The silver-stained gel illustrates final sample purity, while the western blot, probed with the 9E10 antibody recognizing the c-Myc tag, confirms the presence and integrity of scFv NUsc1.

**Fig. S2:**
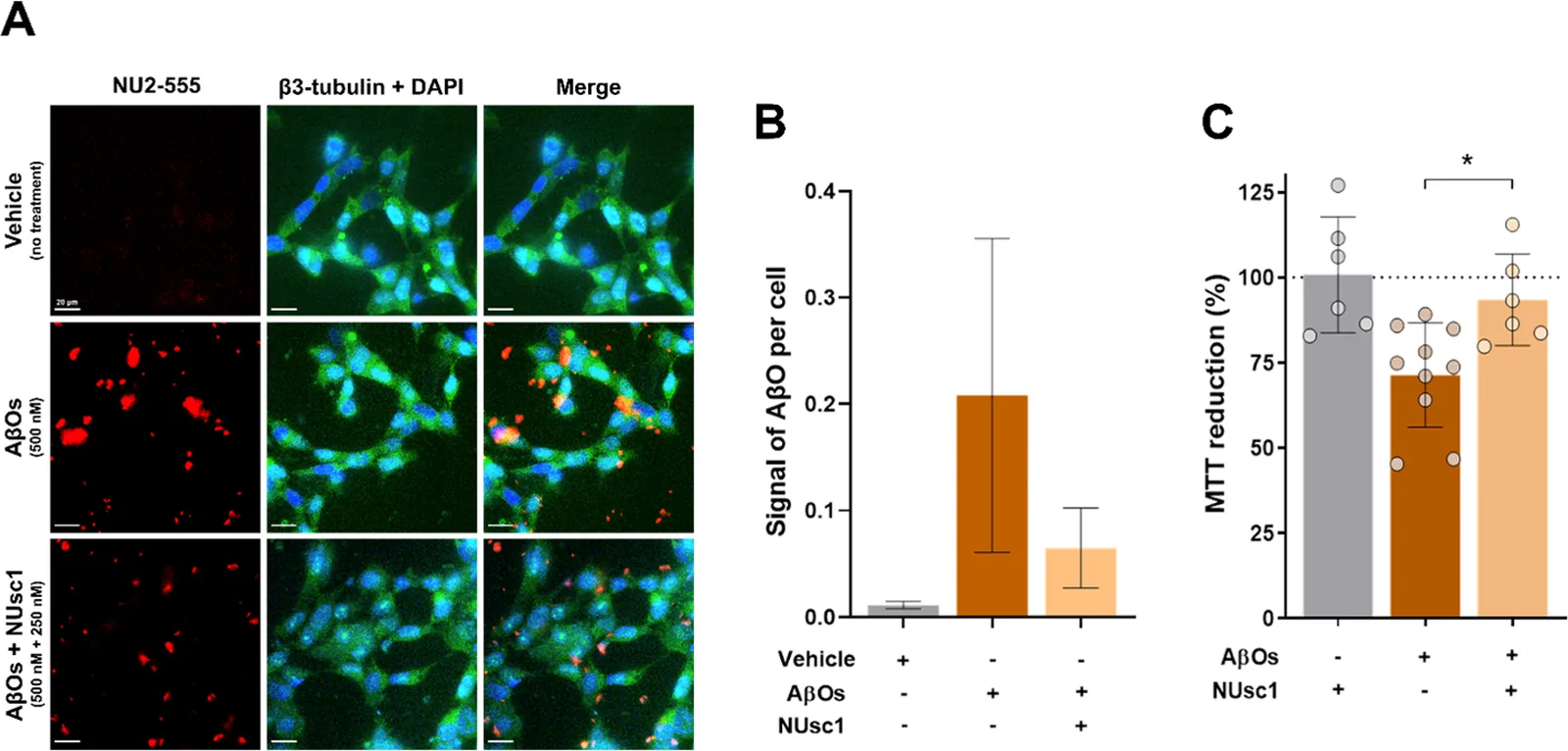
**NUsc1 prevents toxicity of Aβ-oligomers in human neuronal phenotipic cells.** (A) MC-65 human neuronal phenotypic cells were treated with vehicle, Aβ oligomers (AβOs) at 500 nM, or a combination of AβOs and NUsc1 (500 nM + 250 nM) for 4 hours. Following treatment, cells were fixed and immunostained using anti-AβOs antibody NU2-555 and anti-βIII tubulin antibody. Nuclei were stained with DAPI. [B] Quantification of panel A. [C] Cell viability was assessed using an MTT assay on SH-SY5Y cells differentiated into neuronal phenotypes, which were challenged for 24 hours with AβOs, NUsc1, or a mixture of AβOs and NUsc1. The dashed line corresponds to the maximum viability of control wells (non-treated cells). Each experimental condition was performed at least in quadruplicate for each independent experiment. Viability quantification is reported as mean ± SEM of at least 6 independent experiments per condition. Statistical analysis was performed using unpaired Student’s t-test. Asterisks denote p < 0.05 (compared to AβOs-treated cells).

**Fig. S3:**
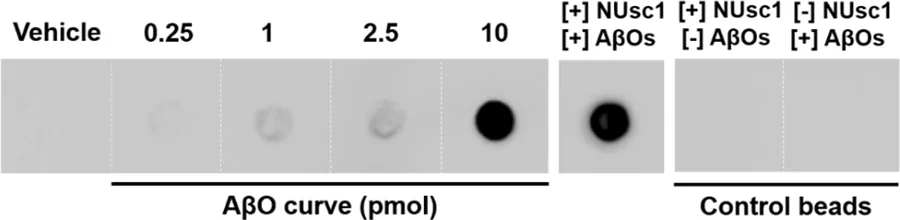
**NUsc1 isolates AβOs using m270 epoxy beads.** A representative Dot blot of eluted samples from synthetic AβOs immunoprecipitated using NUsc1. In addition to the synthetic AβOs curve, negative controls are also shown. Membrane was revealed with α-AβOs NU2 antibody.

**Fig. S4:**
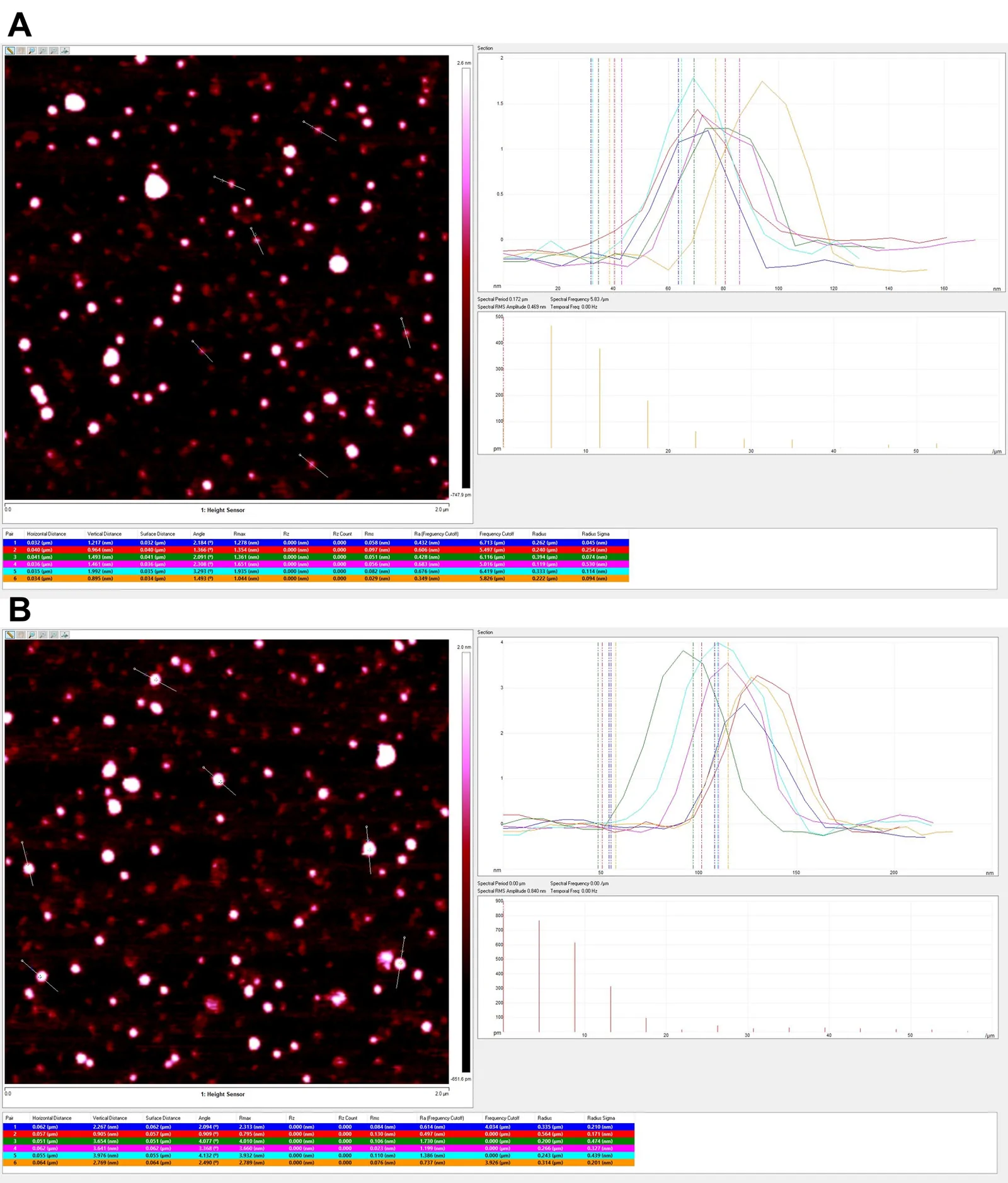
**AFM cross-section analysis.** Representative molecules were selected from each AFM image based on the major population observed. Cross-sections (150–200 nm) of six molecules in (A) NUsc1 and (B) NUsc1-ABO complex were analyzed to capture the surrounding local environment. The vertical scale was kept constant to enable comparison of molecular heights; lateral dimensions were not compared due to differing cross-section lengths.

**Fig. S5:**
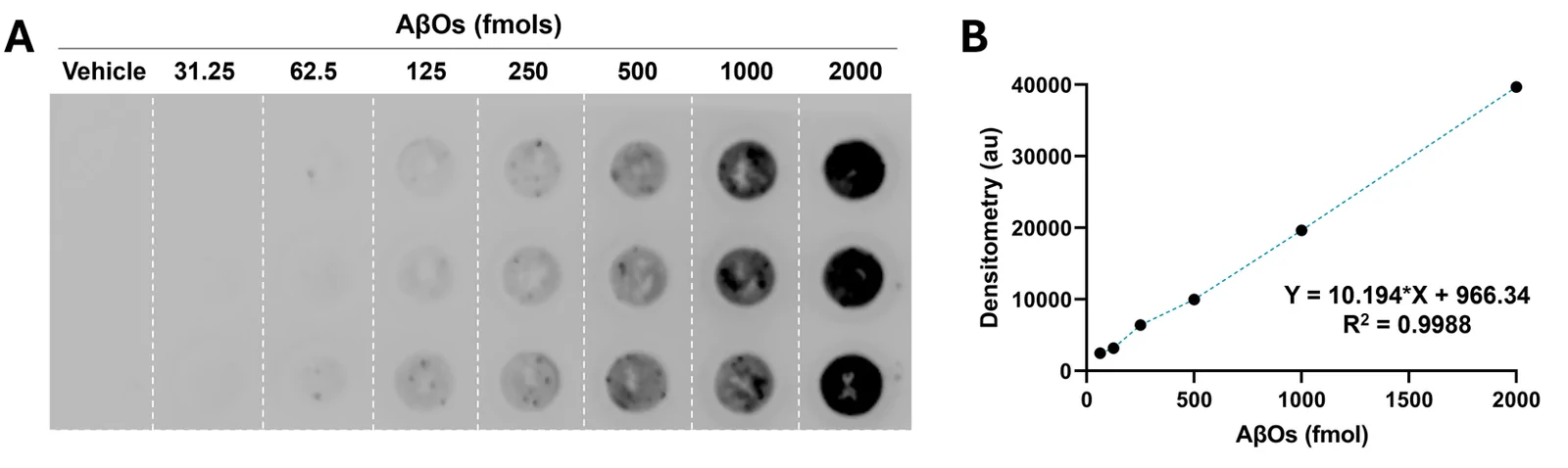
**Standard curve for dot blot analysis of brain-derived AβOs.** Standard curve was generated using synthetic oligomers to enable semi-quantitative analysis of AβO levels in NUsc1-immunoisolated samples. (A) Known amounts of synthetic AβOs were spotted and probed with the α-AβO antibody NU2 under the same conditions as experimental samples. (B) Densitometry measurements were performed using ImageJ, and calibration curve was constructed in GraphPad Prism. This curve was used to estimate relative AβO levels in brain-derived samples shown in Fig. 7.

**Fig. S6:**
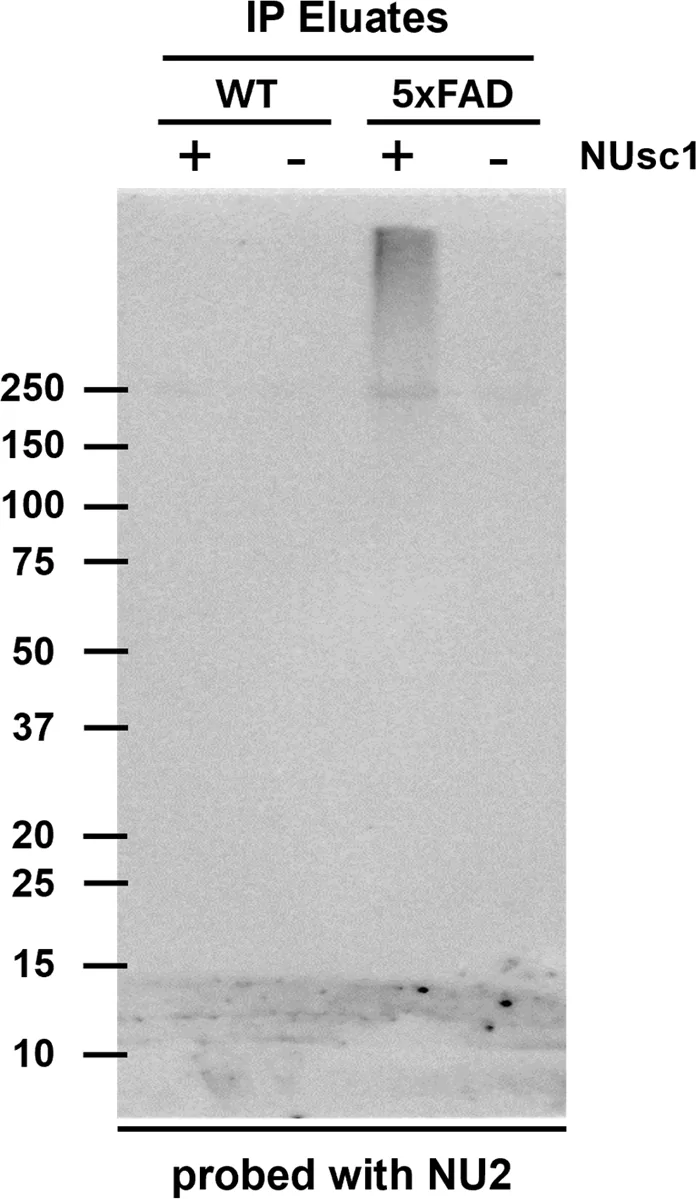
**Western blot of AβOs isolated from 5xFAD mouse brain.** Non-boiled eluates containing isolated brain-extract AβOs were resolved by SDS–PAGE using a 4–20% Tris–glycine gradient gel run at 125 V for approximately 80 minutes. Following electrophoresis, proteins were transferred onto a nitrocellulose membrane and probed with an anti-AβO antibody NU2. For each condition, eluates from five independent 5xFAD mouse brain extracts were pooled to ensure sufficient AβO yield for signal detection. Molecular weight markers (in kDa) are indicated on the left.

**Fig. S7:**
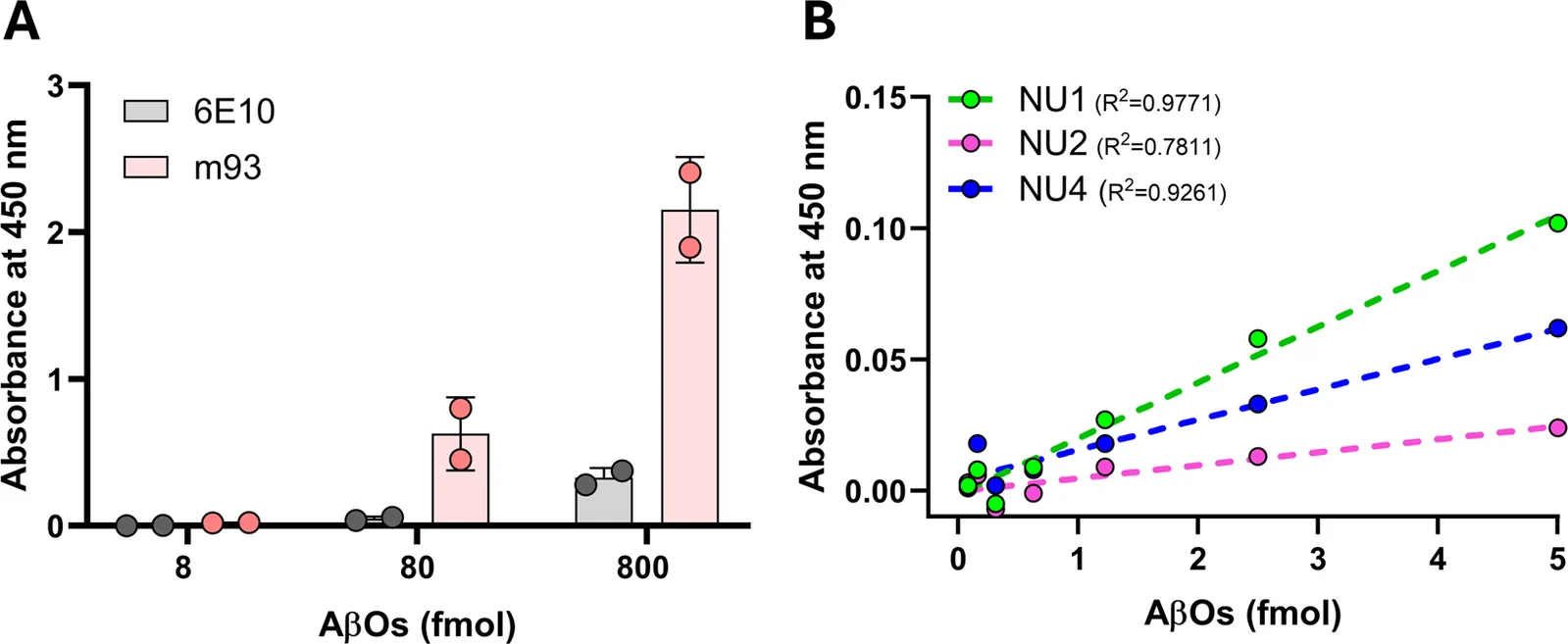
**Comparative detection of AβOs by sandwich ELISA using anti-pan-Aβ or anti-AβO antibodies.** (A) Signal at increasing amounts of AβOs (8, 80, and 800 fmol) using as capture antibodies 6E10 (gray) or M93 (pink). The use of M93 promoted a substantially higher signal intensity than 6E10. Data are shown as mean ± SD. (B) Concentration dependence curves for the same assay, with different NU antibodies as capture antibodies. Linear regression revealed strong correlation for NU1 (R² = 0.9771) and NU4 (R² = 0.9261), whereas NU2 showed reduced linearity (R² = 0.7811) at this lower AβO concentration range.

**Fig. S8:**
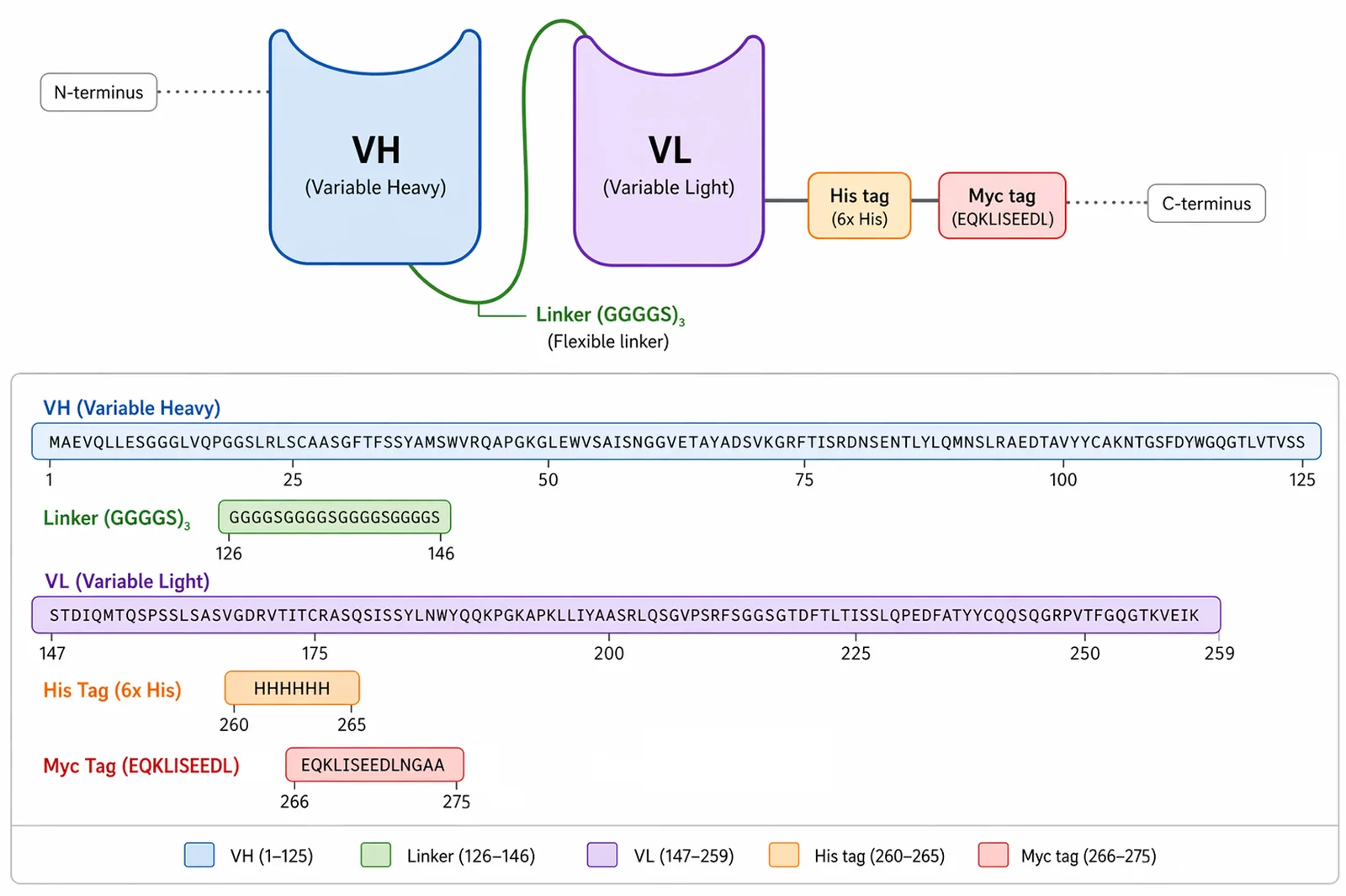
**Schematic representation of the scFv NUsc1.** The NUsc1 single-chain variable fragment (scFv) structure includes the variable heavy (VH) domain, a flexible peptide linker, a variable light (VL) domain, His tag, and Myc tag. Amino acid sequence of NUsc1 with each segment is indicated in the lower panel.
