# Supplementary material for: Use of high-affinity scFv antibody NUsc1 for isolation and sensitive detection of Alzheimer’s-associated amyloid beta oligomers
Source: J Biol Chem. 2026 Aug 26;302(10):113499. doi: 10.1016/j.jbc.2026.113499 (PMC13631620; doi:10.1016/j.jbc.2026.113499)
Supplement: Supplementary file 1 [file mmc1.docx]

## Supplementary information

**Use of high-affinity scFv antibody NUsc1 for isolation and sensitive detection of Alzheimer’s-associated amyloid beta oligomers**

Raquel Maria de Campos^1,2,*^, Mariane Fávero Carraro^2^, André Luiz Brandão Bitencourt^2^, Joshua J. Jerisha^1^, Navid Jubaer Ayon^3^, Vuslat B. Juska^4^, Nathalia Reges Pinheiro^2^, Elizabeth A. Johnson^1^, Raghad N. Nowar^1^, John P. McGee^3^, Mauro Exequiel Alfaro^5^, Maria Victoria Oberholzer^5^, Vanina Grippo^6^, Erika N. Cline^1^, Kirsten L. Viola^1^, Neil L. Kelleher^3^, Martin Habif^5^, Diana Alicia Jerusalinsky^5^, Gajendra S. Shekhawat^7^, Morgan B. Elmor^8^, James J. Lah^8^, Allan I. Levey^8^, William L. Klein^1^, Adriano Sebollela^2^

^1^Department of Neurobiology, Northwestern University, Evanston, Illinois 60208, United States

^2^Department of Biochemistry and Immunology, Ribeirao Preto Medical School, University of São Paulo, SP, 14049-900, Brazil

^3^Departments of Chemistry and Molecular Biosciences, Department of Chemical and Biological Engineering, the Chemistry of Life Processes Institute, the Proteomics Center of Excellence at Northwestern University, Evanston, Illinois 60208, United States

^4^Department of Chemistry, Weinberg College of Arts & Sciences, Northwestern University, Evanston, Illinois 60208, United States

^5^Instituto de Biología Celular y Neurociencia (IBCN), Universidad de Buenos Aires (UBA) and Consejo Nacional de Investigaciones Científicas y Técnicas (CONICET), Ciudad Autónoma de Buenos Aires, Argentina

^6^Instituto de Ciencia y Tecnología “Dr. César Milstein” (ICT-Milstein), Consejo Nacional de Investigaciones Científicas y Técnicas (CONICET) – Fundación Pablo Cassará, Ciudad Autónoma de Buenos Aires, Argentina

^7^International Institute for Nanotechnology and NUANCE Center, Department of Materials Science and Engineering, Northwestern University, Evanston, Illinois 60004, United States

^8^Department of Neurology and Goizueta Alzheimer’s Disease Research Center, Emory University, Atlanta, GA 30329, United States

## Fig S1. NUsc1 production and purification quality control. Representative silver-stained SDS-PAGE gel and western blot membrane showing NUsc1 expression and purification from E. coli HB2151. Samples include cell lysate supernatant, flow-through from Ni²⁺ resin, third wash from Ni²⁺ resin, Ni²⁺ resin eluate, and size-exclusion chromatography (SEC) eluate. NUsc1 corresponds to a protein of approximately 29 kDa. The silver-stained gel illustrates final sample purity, while the western blot, probed with the 9E10 antibody recognizing the c-Myc tag, confirms the presence and integrity of scFv NUsc1.

## Fig S2. NUsc1 prevents toxicity of Aβ-oligomers in human neuronal phenotipic cells. (A) MC-65 human neuronal phenotypic cells were treated with vehicle, Aβ oligomers (AβOs) at 500 nM, or a combination of AβOs and NUsc1 (500 nM + 250 nM) for 4 hours. Following treatment, cells were fixed and immunostained using anti-AβOs antibody NU2-555 and anti-βIII tubulin antibody. Nuclei were stained with DAPI. [B] Quantification of panel A. [C] Cell viability was assessed using an MTT assay on SH-SY5Y cells differentiated into neuronal phenotypes, which were challenged for 24 hours with AβOs, NUsc1, or a mixture of AβOs and NUsc1. The dashed line corresponds to the maximum viability of control wells (non-treated cells). Each experimental condition was performed at least in quadruplicate for each independent experiment. Viability quantification is reported as mean ± SEM of at least 6 independent experiments per condition. Statistical analysis was performed using unpaired Student’s t-test. Asterisks denote p < 0.05 (compared to AβOs-treated cells).

### ***Fig S3.* NUsc1 isolates AβOs using m270 epoxy beads.** A representative Dot blot of eluted samples from synthetic AβOs immunoprecipitated using NUsc1. In addition to the synthetic AβOs curve, negative controls are also shown. Membrane was revealed with α-AβOs NU2 antibody.

### ***Fig S4.* AFM cross-section analysis.** Representative molecules were selected from each AFM image based on the major population observed. Cross-sections (150–200 nm) of six molecules in (A) NUsc1 and (B) NUsc1-ABO complex were analyzed to capture the surrounding local environment. The vertical scale was kept constant to enable comparison of molecular heights; lateral dimensions were not compared due to differing cross-section lengths.

### ***Fig S5.* Standard curve for dot blot analysis of brain-derived AβOs.** Standard curve was generated using synthetic oligomers to enable semi-quantitative analysis of AβO levels in NUsc1-immunoisolated samples. (A) Known amounts of synthetic AβOs were spotted and probed with the α-AβO antibody NU2 under the same conditions as experimental samples. (B) Densitometry measurements were performed using ImageJ, and calibration curve was constructed in GraphPad Prism. This curve was used to estimate relative AβO levels in brain-derived samples shown in Fig. 7.

### ***Fig S6.* Western blot of AβOs isolated from 5xFAD mouse brain.** Non-boiled eluates containing isolated brain-extract AβOs were resolved by SDS–PAGE using a 4–20% Tris–glycine gradient gel run at 125 V for approximately 80 minutes. Following electrophoresis, proteins were transferred onto a nitrocellulose membrane and probed with an anti-AβO antibody NU2. For each condition, eluates from five independent 5xFAD mouse brain extracts were pooled to ensure sufficient AβO yield for signal detection. Molecular weight markers (in kDa) are indicated on the left.

***Fig S7.* Comparative detection of AβOs by sandwich ELISA using anti-pan-Aβ or anti-AβO antibodies.** (A) Signal at increasing amounts of AβOs (8, 80, and 800 fmol) using as capture antibodies 6E10 (gray) or M93 (pink). The use of M93 promoted a substantially higher signal intensity than 6E10. Data are shown as mean ± SD. (B) Concentration dependence curves for the same assay, with different NU antibodies as capture antibodies. Linear regression revealed strong correlation for NU1 (R² = 0.9771) and NU4 (R² = 0.9261), whereas NU2 showed reduced linearity (R² = 0.7811) at this lower AβO concentration range.

***Fig S8.* Schematic representation of the scFv NUsc1.** The NUsc1 single-chain variable fragment (scFv) structure includes the variable heavy (VH) domain, a flexible peptide linker, a variable light (VL) domain, His tag, and Myc tag. Amino acid sequence of NUsc1 with each segment is indicated in the lower panel.

***Table S1. Binding kinetics of scFv NUsc1 to Aβ-oligomers measured by biolayer interferometry (BLI)***

| **Conc.** | **Response** | **K*_D_*** | **K*_D_* error** | **k*_a_*** | **k*_a_* error** | **k*_dis_*** | **k*_dis_* error** | **R*_max_*** | **R*_max_* error** | **k*_obs_*** | **k*_obs_* error** | **R*_eq_*** | **R*_eq_*/R*_max_*** | **Full X^2^** | **Full R^2^** |
| --- | --- | --- | --- | --- | --- | --- | --- | --- | --- | --- | --- | --- | --- | --- | --- |
| ***nm*** | ***nm*** | ***M*** | ***M*** | ***1/M·s*** | ***1/M·s*** | ***1/s*** | ***1/s*** | ***nm*** | ***nm*** | ***1/s*** | ***1/s*** | ***Nm*** | ***%*** |  |  |
| 500 | 0.5541 | 8.87×10⁻⁹ | 4.51×10⁻¹¹ | 2.97×10⁴ | 8.41×10¹ | 2.63×10⁻⁴ | 1.11×10⁻⁶ | 0.5172 | 0.0003 | 1.51×10⁻² | 4.32×10⁻⁵ | 0.5081 | 98.3 | 13.6153 | 0.9848 |
| 250 | 0.3369 | 8.87×10⁻⁹ | 4.51×10⁻¹¹ | 2.97×10⁴ | 8.41×10¹ | 2.63×10⁻⁴ | 1.11×10⁻⁶ | 0.342 | 0.0003 | 7.69×10⁻³ | 2.21×10⁻⁵ | 0.3303 | 96.6 | 13.6153 | 0.9848 |
| 125 | 0.2876 | 8.87×10⁻⁹ | 4.51×10⁻¹¹ | 2.97×10⁴ | 8.41×10¹ | 2.63×10⁻⁴ | 1.11×10⁻⁶ | 0.3231 | 0.0004 | 3.98×10⁻³ | 1.16×10⁻⁵ | 0.3017 | 93.4 | 13.6153 | 0.9848 |
| 62.5 | 0.1781 | 8.87×10⁻⁹ | 4.51×10⁻¹¹ | 2.97×10⁴ | 8.41×10¹ | 2.63×10⁻⁴ | 1.11×10⁻⁶ | 0.2588 | 0.0005 | 2.12×10⁻³ | 6.37×10⁻⁶ | 0.2266 | 87.6 | 13.6153 | 0.9848 |
| 31.3 | 0.0883 | 8.87×10⁻⁹ | 4.51×10⁻¹¹ | 2.97×10⁴ | 8.41×10¹ | 2.63×10⁻⁴ | 1.11×10⁻⁶ | 0.1959 | 0.0006 | 1.19×10⁻³ | 3.74×10⁻⁶ | 0.1526 | 77.9 | 13.6153 | 0.9848 |

Kinetic parameters for scFv NUsc1 binding to Aβ oligomers (AβOs) were determined by BLI in Ham’s F-12 buffer. Biotinylated NUsc1 was immobilized on streptavidin (SAX) biosensors, and analyte concentrations ranged from 31.3 to 500 nM, each measured in at least duplicate. Sensorgrams were fit to a 1:1 Langmuir binding model to obtain association (ka) and dissociation (kdis) rate constants, and KD was calculated as kdis/ka.

### ***Table S2*. Demographic characteristics of study participants**

| Group | GUID | Gender | Race | Age | Cohort | Education |
| --- | --- | --- | --- | --- | --- | --- |
| CONTROL | 1.49917 | Female | White | 61.6 | EHBS | 15 |
| CONTROL | 1.51316 | Female | White | 66.0 | EHBS | 18 |
| CONTROL | 1.48625 | Female | White | 72.1 | EHBS | 16 |
| CONTROL | 1.40225 | Female | White | 78.2 | ADRC | 18 |
| CONTROL | 1.45587 | Female | White | 74.5 | ADRC | 16 |
| CONTROL | 1.48349 | Male | White | 75.6 | ADRC-EHBS | 20 |
| CONTROL | 1.59345 | Male | White | 66.1 | EHBS | 13 |
| CONTROL | 1.40545 | Male | White | 80.8 | ADRC | 19 |
| CONTROL | 1.58545 | Male | White | 64.7 | EHBS | 20 |
| CONTROL | 1.58015 | Male | White | 62.9 | EHBS | 16 |
| AD | 1.89147 | Female | White | 73.1 | COGNITIVE | 12 |
| AD | 1.89420 | Female | White | 78.6 | COGNITIVE | 16 |
| AD | 1.59573 | Female | White | 66.0 | COGNITIVE | 16 |
| AD | 1.93398 | Female | White | 60.9 | COGNITIVE | 12 |
| AD | 1.95394 | Female | White | 75.5 | COGNITIVE | 14 |
| AD | 1.90914 | Male | White | 64.4 | COGNITIVE | 16 |
| AD | 1.90766 | Male | White | 80.1 | COGNITIVE | 20 |
| AD | 1.93649 | Male | White | 61.6 | COGNITIVE | 16 |
| AD | 1.96215 | Male | White | 66.4 | COGNITIVE | 16 |
| AD | 1.94279 | Male | White | 75.7 | COGNITIVE | 18 |

***Table S3*. Clinical scores, APOE genotypes, and cerebrospinal fluid biomarkers (Aβ42, total Tau, and pTau) of participants**

| Group | GUID | APOE | MoCA | Aβ42 | tTau | pTau | tTau:Aβ42 | pTau:Aβ42 |
| --- | --- | --- | --- | --- | --- | --- | --- | --- |
| CONTROL | 1.49917 | e3/e3 | 30 | 975.50 | 120.60 | 9.58 | 0.124 | 0.0098 |
| CONTROL | 1.51316 | e3/e3 | 28 | 2302.00 | 198.70 | 15.59 | 0.086 | 0.0068 |
| CONTROL | 1.48625 | e3/e3 | 20 | 1227.00 | 152.00 | 12.00 | 0.124 | 0.0098 |
| CONTROL | 1.40225 | e2/e3 | 30 | 2500.00 | 220.00 | 19.89 | 0.088 | 0.0080 |
| CONTROL | 1.45587 | e3/e3 | 26 | 2049.00 | 241.40 | 20.71 | 0.118 | 0.0101 |
| CONTROL | 1.48349 | e3/e3 | 30 | 2093.00 | 171.70 | 14.48 | 0.082 | 0.0069 |
| CONTROL | 1.59345 | e2/e3 | 25 | 1431.00 | 174.80 | 13.81 | 0.122 | 0.0097 |
| CONTROL | 1.40545 | e3/e3 | 29 | 1212.00 | 158.80 | 14.77 | 0.131 | 0.0122 |
| CONTROL | 1.58545 | e3/e3 | 27 | 1381.00 | 176.40 | 14.88 | 0.128 | 0.0108 |
| CONTROL | 1.58015 | e3/e3 | 25 | 2380.00 | 264.20 | 24.18 | 0.111 | 0.0102 |
| AD | 1.89147 | e3/e4 | 18 | 333.60 | 283.00 | 30.54 | 0.848 | 0.0915 |
| AD | 1.89420 | e3/e3 | 24 | 615.70 | 753.80 | 77.60 | 1.224 | 0.1260 |
| AD | 1.59573 | e3/e4 | 23 | 871.80 | 291.20 | 30.28 | 0.334 | 0.0347 |
| AD | 1.93398 | e4/e4 | 17 | 772.50 | 439.60 | 43.04 | 0.569 | 0.0557 |
| AD | 1.95394 | e3/e3 | 16 | 337.90 | 212.20 | 20.37 | 0.628 | 0.0603 |
| AD | 1.90914 | e3/e3 | 14 | 456.20 | 187.00 | 19.58 | 0.410 | 0.0429 |
| AD | 1.90766 | e3/e4 | 22 | 572.80 | 221.20 | 23.06 | 0.386 | 0.0403 |
| AD | 1.93649 | e3/e4 | 22 | 599.00 | 434.30 | 45.37 | 0.725 | 0.0757 |
| AD | 1.96215 | e3/e3 | 18 | 783.50 | 473.00 | 49.78 | 0.604 | 0.0635 |
| AD | 1.94279 | e3/e4 | 18 | 717.40 | 264.90 | 24.71 | 0.369 | 0.0344 |
